# Bulk Perovskite Crystal Properties Determined by Heterogeneous Nucleation and Growth

**DOI:** 10.3390/ma16052110

**Published:** 2023-03-05

**Authors:** Pranta Barua, Inchan Hwang

**Affiliations:** Department of Electronic Materials Engineering, Kwangwoon University, Seoul 01897, Republic of Korea

**Keywords:** nucleation, crystal growth, perovskite, Gibbs free energy, bulk crystallinity

## Abstract

In metal halide perovskites, charge transport in the bulk of the films is influenced by trapping and release and nonradiative recombination at ionic and crystal defects. Thus, mitigating the formation of defects during the synthesis process of perovskites from precursors is required for better device performance. An in-depth understanding of the nucleation and growth mechanisms of perovskite layers is crucial for the successful solution processing of organic–inorganic perovskite thin films for optoelectronic applications. In particular, heterogeneous nucleation, which occurs at the interface, must be understood in detail, as it has an effect on the bulk properties of perovskites. This review presents a detailed discussion on the controlled nucleation and growth kinetics of interfacial perovskite crystal growth. Heterogeneous nucleation kinetics can be controlled by modifying the perovskite solution and the interfacial properties of perovskites adjacent to the underlaying layer and to the air interface. As factors influencing the nucleation kinetics, the effects of surface energy, interfacial engineering, polymer additives, solution concentration, antisolvents, and temperature are discussed. The importance of the nucleation and crystal growth of single-crystal, nanocrystal, and quasi-two-dimensional perovskites is also discussed with respect to the crystallographic orientation.

## 1. Introduction

Advances in thin-film perovskite solar cells (PSCs) and perovskite light-emitting diodes (PeLEDs) have extensively focused on the development of perovskite crystallinity with a lower density of traps that act as nonradiative recombination centers. The power conversion efficiency (PCE) of PSCs has rapidly improved from 3.8% to 25.8% since their introduction in 2009 [1,2,3,4], while the external quantum efficiency (EQE) of PeLEDs in light-emitting applications has remarkably increased to >20% from 1%, which was recorded in 2014 [5,6]. Perovskite materials exhibit better optoelectronic properties than other hybrid functional materials [7,8,9]. The general formula of metal halide perovskite (MHP) is defined as ABX_3_, where A = CH_3_NH_3_^+^ (MA), HC(NH_2_)_2_^+^ (FA), or Cs^+^, B = Pb^2+^ or Sn^2+^, and X = I^−^, Cl^−^, or Br^−^. Perovskites have sparked significant interest among researchers and industrial experts owing to their outstanding optoelectronic features, such as high charge carrier mobilities, low exciton binding energies, long carrier diffusion lengths, exceptional defect tolerance, ambipolar charge transport, flexible tunability, and high light absorption coefficients, as well as their easy wavelength-tuning abilities [6,10,11,12].

The unique intrinsic properties of MHPs lead to excellent optoelectronic properties, which are associated with the band structure. The position of the valence band maximum (VBM) and conduction band minimum (CBM) can be finely tuned by substituting B and/or X elements because the band structure is determined by these elements [13]. The conduction band minimum is determined predominantly by Pb 6p orbitals, with small contributions from I 5p orbitals, while the valence band maximum is formed by the strong antibonding coupling between Pb 6s and I 5p orbitals [14]. For optical absorption, the symmetry in the perovskite crystal structure leads to a direct bandgap because of a direct p-p electronic transition between Pb 6p conduction states and I 5p valence states, enabled by the lone-pair electrons in Pb s orbitals, allowing high optical absorption [15]. The high mobility of charge carriers is attributed to the small effective masses of both electrons and holes, leading to an ambipolar character [16]. This is a distinguishable feature from most other semiconductors, which exhibit unbalanced charge transport due to differences in the effective masses of electrons and holes. The balanced effective mass can be explained by the presence of lone-pair electrons in Pb s orbitals. The strong interactions between s and p orbitals near the valence band maximum make the upper valence band states more dispersive, which leads to small hole effective mass, comparable to that of electrons [17]. Defect tolerance is another exceptional property of MHPs. The defect levels in halide perovskites are typically located near the band edges rather than in the middle of the bandgap. This implies that they are unlikely to act as nonradiative recombination centers. Therefore, the concept of “defect tolerance” has been used to explain the high efficiency of halide perovskites [18,19]. A long diffusion length is the consequence of high defect tolerance and high charge carrier mobility [20]. 

Defect formation in perovskite layers cannot be avoided because they are synthesized from precursors such as organic or inorganic halides and metal halides [21,22]. Perovskites have shallow and deep traps depending on the kinds of defects, whether they are in the form of interstitials, anti-sites, or vacancies in each perovskite component [23,24,25]. Shallow traps are inactive for the nonradiative recombination of charge carriers, so perovskites with particular structures are often considered to be defect-tolerant [26,27,28,29,30]. However, deep traps are centers for nonradiative recombination, which is detrimental to device performance. Several approaches have been proposed to address this drawback, and they can be classified into two categories depending on how nucleation and growth kinetics are controlled [31,32,33,34,35,36,37] to eliminate crystal defects: mainly from the surface or from the bulk of perovskites. Defects generated during crystal nucleation and growth change the perovskite lattice microstrain, which can be further degraded by environmental factors such as humidity, temperature, and photon irradiation [38,39]. Because of interdependent material properties, perovskites possess a complex framework, and the complexity of this framework is intensified by ion-migration-induced phase segregation, which causes perovskite film degradation [25,34,40,41,42,43,44]. A novel conjugated polyelectrolyte polymer, PHIA, was introduced for its incorporation into MAPbI_3_ perovskite solar cells to passivate defects [45]. The PHIA polymer provided several advantageous properties, including the ability of the anchoring groups to interact with various defects in perovskites and decrease the number of trap states. PHIA passivation enhanced the long-term stability of perovskite solar cells by increasing the hydrophobicity of the perovskite layer. Chao et al. [46] proposed ionic liquids as emerging solvents for PSCs. The ionic liquids included MAAc, MAFa, BAAc, etc., which are green, pollution-free, and low-cost. These substances can be applied for the improvement of ionic-liquid-based PSCs via interfacial engineering, solvent engineering, and additive engineering for defect passivation. 

Grain size matters, but in the opposite way, to realize high-performance PSCs and PeLEDs. For PSCs, a bigger grain size is desired so as to have a low area of grain boundaries for fewer defects. In contrast, small grains benefit electroluminescence in PeLEDs to confine excitons with a high binding energy [47,48,49,50]. Thus, realizing controlled crystal growth according to specific applications is a considerable challenge. The growth of a perovskite crystal depends on several key parameters, such as the nucleation density, Gibbs free energy, lattice constant, and so on [38,51,52,53]. Detailed analyses of the growth parameters and conditions, as well as their variations, from the onset of crystal growth are essential for determining the bulk crystallinity of perovskites. In this review, we first discuss the nucleation process, its correlation with Gibbs free energy, and the distinctions between homogeneous and heterogeneous nucleation. Next, the role of heterogeneous nucleation in perovskite crystal formation is discussed in detail, followed by a brief description of the lattice constant, including the impact of interlayer and bulk crystallinity alterations. The influence of grain size on interfacial crystal quality is elucidated, along with the underlying mechanisms of the polymer-assisted crystallization and heterogeneous nucleation of quasi-two-dimensional (2D), single-crystal, and nanocrystal perovskites. Finally, the impact of temperature on crystallization and nucleation processes is discussed.

## 2. Nucleation and Gibbs Free Energy

A nucleus is an initial cluster of molecules or atoms that starts a new phase. Nucleation is the process in which a specific thermodynamic phase functions as a template for the irreversible event that subsequently occurs, which is crystal growth within the medium [54,55]. For the classical model of nucleation, a cluster of solute molecules begins to form from the supersaturated solution, subsequently promoting the growth of crystalline nuclei, as the atoms from the liquid are attached to the nuclei until they are used up, to form a solid crystal. In perovskite precursor solutions, as the solvent evaporates in the spin-coating process, the free energy is modified when the temporal solution concentration varies in supersaturation, leading to the formation of a new phase composed of the parent precursors via crystal growth. The supersaturation condition initiates nucleation, producing nuclei clusters that are favorable to subsequent crystal growth. For nuclei to stably form without going backward to dissolution, they should overcome the activation energy barrier, which corresponds to the maximum of the change in Gibbs free energy [56]. It is an endergonic reaction that requires external energy to be driven if the Gibbs free energy increases, Δ*G* > 0, and thus does not occur in a spontaneous manner. In contrast, if the Gibbs free energy decreases, Δ*G* < 0, the reaction takes place spontaneously and is called an exergonic reaction. In thermodynamic equilibrium, there is no change in Gibbs free energy (Δ*G* = 0). 

Depending on the location of where nuclei are generated, two types of nucleation are classified: homogeneous and heterogeneous nucleation. For homogeneous nucleation, which occurs in the interior of liquid states, a spherical shape is assumed for nuclei, with a radius *r*. Changes in volume (Δ*G_v_*) and surface *(*Δ*G_s_*) free energy contribute to the change in the total Gibbs free energy (Δ*G_total_*). Δ*G_v_* is the Gibbs free energy change for solidification. Under circumstances that meet the conditions for solidification, the solid state has a lower free energy than the liquid state. That is, in those cases, Δ*G_v_* is always negative and is the product of the specific Gibbs free energy (Δg) and the volume of the sphere assumed for nuclei inside of liquids. Δ*G_s_* is the Gibbs free energy change resulting from the formation of the boundary between the liquid and the solidified nuclei. This value is always positive regardless of the conditions for solidification and is obtained by the product of the specific surface energy (γ) and the surface area of the nucleus sphere. Therefore, Δ*G_total_* is expressed by the following equation [52]:

(1) $$\Delta G_{total}=\Delta G_{s}+\Delta G_{v}=4\pi r^{2}\gamma +\frac{4}{3}\pi r^{3}\Delta g$$

**Figure 1:**
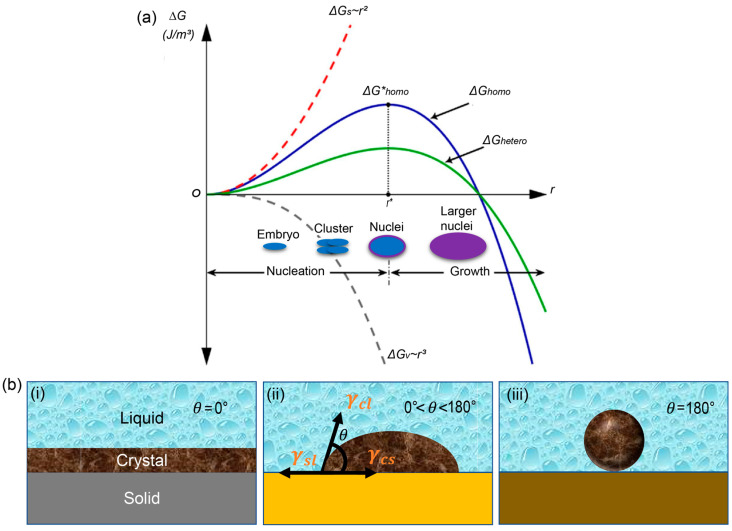
Nucleation and Gibbs free energy. (**a**) Illustration of changes in Gibbs free energy as a function of the particle radius, *r,* where changes in Gibbs free energy for surface, volume, homogeneous nucleation, and heterogeneous nucleation are denoted as Δ*G_s_*, Δ*G_v_*, Δ*G_homo_*, and Δ*G_hetero_*, respectively [58]. (**b**) Schematic of heterogeneous nucleation on a solid with different contact angles; (**i**) *θ* = 0°, (**ii**) 0° < *θ* < 180°, and (**iii**) *θ* = 180° [59]. Reprinted with permission.

Figure 1a illustrates the surface free energy and volume free energy as a function of the radius of the nucleus. As Δ*G_v_* is proportional to *r*^3^ and Δ*G_s_* is proportional to *r*^2^, Δ*G_total_* increases with *r*, and it monotonically decreases due to the contribution of Δ*G_v_* at high *r* values, followed by the maximum point. The maximum of Δ*G_total_* is the activation energy that must be overcome for nucleation to begin and for growth to proceed toward a solid state. The corresponding radius at which *d*Δ*G_total_/dr* = 0 is referred to as the critical radius, r*.

Although we have used the term “nuclei” for convenience, the term should be used carefully, as it differs depending on the radius relative to the critical radius. If the radius is greater than the critical radius, the solidified particles are referred to as nuclei. The nuclei can sustain in the solution rather than redissolving in the solution and thus can grow further toward the path of nucleation, because the free energy decreases as the nuclei grow. In contrast, if the radius of the solidified particles is smaller than the critical radius, they are called embryos or clusters. They redissolve in the solution, as the free energy is lowered when the radius becomes smaller. Therefore, r* can be considered the lowest radius at which a nucleus can exist in the solution and grow further without redissolving in it, and it can be derived as follows [51]:

(2) $$r*=\frac{-2\gamma }{\Delta g}$$

Equation (2) indicates that the critical radius of a nucleus is determined by the ratio of the specific surface energy change to the specific volume free energy change. This suggests that the probability of nucleation can be controlled by modulating the critical radius, which is determined by the ratio of the two free energy changes. The illustration shown at the bottom of Figure 1a depicts the different statuses of solidified particles in the liquid state depending on their radius. 

So far, we have discussed the generalized theory of nucleation, including the phase transition from a liquid to a solid. For perovskites, the change in Gibbs free energy should be associated with the solidification of solutes in the solution, which occurs when it meets the conditions for supersaturation. The degree of supersaturation can be defined as *S* = *C/Cs*, where *C* is the solute concentration, and *Cs* is the solubility limit (*C* > *Cs*), and thus, Δ*g* can be expressed as follows [57]:

(3) $$\Delta g=-\frac{k_{B}T\;ln\left(S\right)}{v}$$

where *T* is the temperature, *k*_B_ is the Boltzmann constant, and *v* is the molar volume. Equation (3) provides practical information on the parameters that need to be controlled in order to facilitate nucleation. To raise the probability of the formation of nuclei, Δ*g* must increase. One method is to increase the solution concentration to reach the supersaturation state earlier during spin coating. In addition, antisolvent dripping during spin coating assists nucleation by intentionally washing the solvent away to facilitate nucleation and thus instantly create many nuclei to form compact morphologies without pinholes in the film. Another method is to increase the temperature. The temperatures of the solution, substrate, atmosphere, and antisolvent are key parameters significantly affecting the free energy and, consequently, nucleation. Therefore, perovskite processing must be carefully conducted considering all of the factors associated with the nucleation process.

### 2.1. Homogeneous and Heterogeneous Nucleation

Homogenous nucleation does not require preferred locations if they occur within the liquid. It is an intrinsic process of materials in which only the material itself that undergoes crystallization is involved. In contrast, heterogeneous nucleation occurs at interfaces. Therefore, the process occurs differently depending on the properties of the interface with other materials. The distinct feature compared to homogeneous nucleation is the shape of the nuclei. As the nuclei cannot be considered to be perfect spheres, the deformed nucleus shape at interfaces can lower the Gibbs free energy barrier. In real experiments, the solution phases contain impurities (bubbles, walls, impurities, etc.), which act as active centers or preferential locations for heterogeneous nucleation. These impurities help in overcoming (lowering) the energy barrier to induce nucleation. The number of nuclei produced at the interface with a substrate is controlled by its relevant nucleation kinetics. The barrier of the change in free energy for heterogeneous nucleation is different from those expected for ideal spherical particles because the nucleus shape differs from a sphere at the interface. The more deformed geometry results in a lower energy barrier, leading to a higher probability of nucleation and thus the formation of more nuclei. The wettability of the film surface obtained by lowering the energy barrier determines the feasibility of nucleation. Quantitatively, the relationship between the Gibbs free energies of homogeneous and heterogeneous nucleation can be expressed as follows [51]:

(4) $$\Delta G^{*}{}_{hetero}=\Delta G^{*}{}_{homo}.f\left(\theta \right)$$

where θ is the contact angle of the solution with the solid surface, and

(5) $$f\left(\theta \right)=\frac{2-3cos\theta +cos^{3}\theta }{4}\;$$

The contact angle, which is defined as the angle where the liquid, vapor, or crystallization phase interacts with the solid surface, is driven by the relationship between the surface energies/tensions at each interface: the surface tension between the solid and liquid ($\gamma_{sl}$), the solid and crystalline phases ($\gamma_{cs}$), and the liquid and crystalline phases ($\gamma_{cl}$). From Equation (5), it is clear that f*(*θ) is solely dependent on *cos* θ, which is expressed by Young’s equation [59]:

(6) $$\cos\;\theta =\frac{\gamma_{cs}-\gamma_{sl}}{\gamma_{cl}}$$

Higher surface wettability is ensured when the surface energy of the solid surface is higher than the liquid’s surface tension, which keeps the liquid in droplet form. A higher surface energy of the solid surface creates a strong adhesive force that overpowers the surface tension of the liquid and thus spreads over the solid surface. That is, if the surface energy of the solid surface is the highest among the three surface energies, which has super-hydrophilic properties, then the molecular unit of the solution precipitates on the solid surface first, and thus, the contact angle tends to be zero (Figure 1bi). If the surface energy for the crystalline phase is higher than those of the solid and liquid phases, the solid surface cannot fully deliver the driving energy required for the heterogeneous nucleation process. Then, the contact angle will be between 0 and 180° (Figure 1bii). If the surface energy for the solid is lower than those of both the crystalline and liquid phases, then the contact angle tends to 180°, as illustrated in Figure 1biii, which enables nuclei to form a spherical shape. Thus, the free energy of heterogeneous nucleation is equal to that of its homogeneous counterpart. Therefore, the free energy associated with heterogeneous nucleation can be modified by interfacial engineering that changes the contact angle. The wettability of the surface facilitates nucleation at θ < 180° as it lowers the free energy barrier based on Equations (4)–(6). 

### 2.2. Effect of Heterogeneous Nucleation on the Formation of Perovskite Crystals

**Figure 2:**
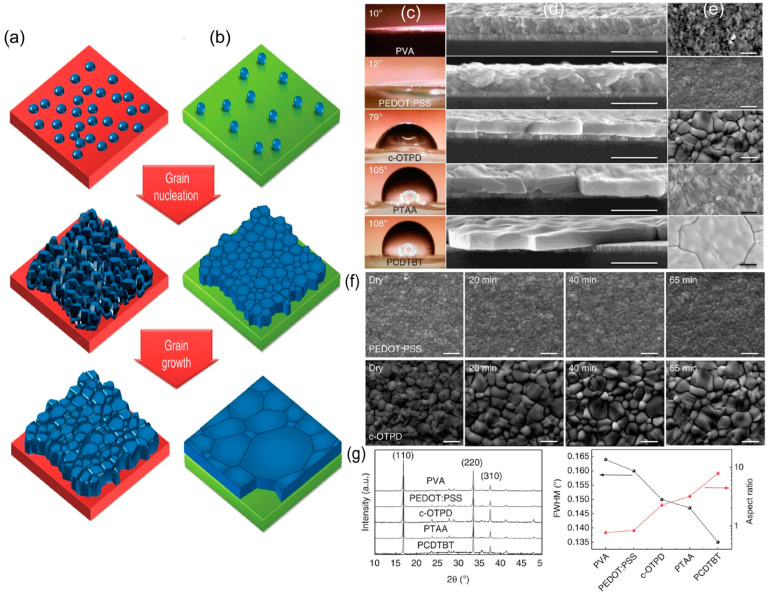
Schematic illustrations of nucleation and grain growth on (**a**) hydrophilic and (**b**) hydrophobic HTLs; (**c**) phenomena of contact angles of water with different HTLs; (**d**) cross-section and top-view SEM images with scale bars, 1 µm; (**e**) top-view SEM images of CH_3_NH_3_PbI_3_ with PEDOT:PSS and c-OTPD as HTLs after drying and with different annealing times (scale bar 1 µm); (**f**) top-view SEM images of PEDOT:PSS and c-OTPD with different annealing time (**g**) X-ray diffraction patterns of the CH_3_NH_3_PbI_3_ perovskites grown on different charge transport layers, and their full-width at half-maximum for the (110) peak (black curve) and the aspect ratio of the average grain size to the thickness [68]. Reprinted with permission.

Given the same supersaturation, a lower activation energy barrier enables heterogeneous nucleation to occur more readily and produce a dense perovskite film with more nuclei and fewer pinholes, which can be achieved by having more deformed nuclei at the interface. Several research groups have explored this strategy to improve surface wettability in order to improve the film morphology [60,61,62,63,64,65,66,67]. Figure 2 presents the impact of hydrophilic and hydrophobic hole transport layers (HTLs) on their corresponding SEM images and XRD patterns [68]. PVA and PEDOT:PSS have wetting surfaces due to their hydroxyl groups, whereas other polymers are hydrophobic. Because c-OTPD has an oxygen group, it is less hydrophobic than PTAA or PCDTBT. The water contact angles for PVA, PEDOT:PSS, c-OTPD, PTAA, and PCDTBT are 10°, 12°, 79°, 105°, and 108°, respectively, as shown in Figure 2c. The measured contact angle of a water droplet represents the wetting capability of polar solvents such as N,N-dimethylformamide (DMF). The cross-sectional and top-view SEM images (Figure 2d,e) exhibit a correlation between the surface wettability and grain morphology. The grain size is larger for hydrophobic HTLS in MAPbI_3_ perovskite films. Figure 2g shows that the perovskite films on hydrophobic HTLs exhibited a stronger and sharper X-ray diffraction pattern, indicating the absence of small grains and an improvement in the crystallinity of the film. In the MAPbI_3_ films on non-wetting HTLs, the majority of the grains were significantly larger than the thickness of the film. The average ratio of grain size to film thickness was 2.3, 3.2, and 7.9 for c-OTPD, PTAA, and PCDTBT, respectively. Gebrimichel et al. [60] utilized different formulation ratios of PEDOT:PSS (i.e., poly(3,4-ethylenedioxythiophene) polystyrene sulfonate) to observe the changes in the contact angle and describe the surface wettability of a perovskite surface. They found that the contact angle can be modified by mixing different chemicals (PH:PH1000, PH:AI4083, PH:PH1000:AI4083), leading to changes in the grain size. Li et al. [63] used two ammonium salts, 4-hydroxyphenethylammoniumiodide (HO-PEAI) and 2-thiopheneethylammonium iodide (2-TEAI), to modify the surface properties of substrates and compared their wettability. The corresponding changes in the contact angle revealed that the hydrophobic organic moieties present in these two ammonium salts (modulators) increased the contact angle of the modulated device compared to that of the pristine one. 

Substrate preheating helps in increasing the number of nucleation sites, as it increases the specific free energy because of high temperature according to Equation (3). Without substrate preheating, the solvent evaporation rate is low, which leads to the slow kinetics of nucleation and crystal growth [69,70,71]. Three-dimensional inorganic CsPbIBr_2_ perovskite films [69] were prepared using preheated substrates, and they were annealed at different temperatures. As the substrate preheating temperature increases, nucleation and crystal growth are facilitated, whereas the number of voids is reduced. For the triple-cation mixture Cs_0.21_FA_0.56_MA_0.23_(I_0.98_Br_0.02_) [70] via a two-step spin-coating process, it was found that the grain size and density of the PbI_2_ crystals could be successfully controlled by modulating the substrate heating temperature, which led to a remarkable improvement in the morphology of the PbI_2_ film upon substrate heating. They observed that with substrate preheating, the morphology of PbI_2_ was significantly improved. Upon increasing the heating temperature, the PbI_2_ films became compact and dense with small grains. This is the consequence of an accelerated rate of solvent evaporation with increasing substrate temperature that enhances the degree of supersaturation of PbI_2_ films. Hui et al. [71] demonstrated that the surface of a TiO_2_ film is only partially covered by CH_3_NH_3_PbI_3_ perovskites on a substrate without preheating. They reported the presence of numerous voids and cracks in the PbI_2_ layer due to the low nucleation density mediated by the slow kinetics of heterogeneous nucleation. In contrast, substrate preheating allows the PbI_2_ nucleation rate to be increased, leading to an increase in the number of nucleation sites. Srivastava et al. [72], using CH_3_NH_3_PbI_3_ perovskite, also reported that before spin coating, changes in temperature between room temperature and 120 °C were found to significantly alter the film morphology. 

The hot-casting technique or substrate heating during perovskite film processing has been developed to produce high-quality perovskite thin films. It was demonstrated that a high substrate temperature can lead to an increase in the thickness of perovskite films, which was explained by a higher density of heterogeneous nuclei, followed by perovskite crystal growth [73,74,75]. This method offers significant advantages, including fast crystallization, a shortened film formation process, a substantial increase in grain size, preferred crystalline orientation, and reduced defect states. According to a study by Chang and colleagues [76], raising the substrate temperature to above 100 °C alters the formation mechanism of MAPbI_3−x_Cl_x_ films. This change leads to a direct formation mechanism, which results in larger grains and, consequently, a PCE improvement. The formation mechanism without heating the substrate requires multiple steps to form perovskites, but the shift to a direct mechanism with a higher substrate temperature was found to be advantageous for achieving better-quality films. In addition, by increasing the evaporation rate during the spin-coating process, this method can effectively decrease the amount of residual solvent [77,78,79].

**Figure 3:**
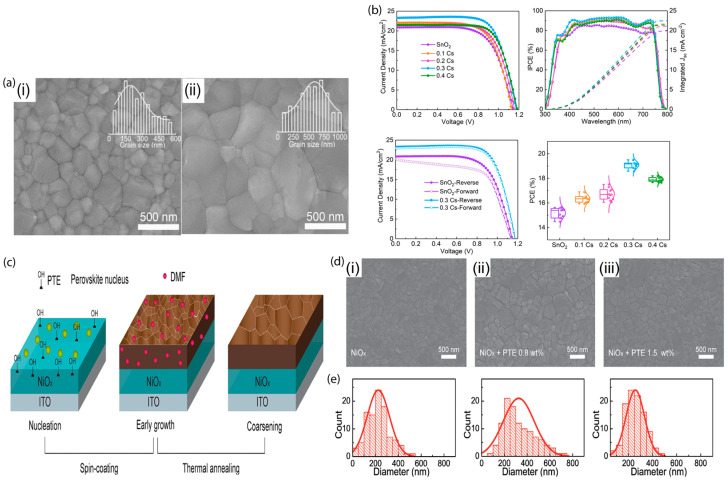
Correlation between grain size change and heterogeneous nucleation kinetics. Top-view SEM images of the perovskite films based on (**a**) (**i**) pristine and (**ii**) 0.3 Cs-treated SnO_2_ films (scale bar is 500 nm). The insets show the statistical grain size distribution. (**b**) Device performance for different concentration of Cs doping in SnO_2_ [80]. (**c**) Schematic diagram presenting nucleation and crystal growth via spin coating and thermal annealing process. (**d**) Top-view SEM images (scale bar is 500 nm) for MAPbI_3_ with (**i**) pristine NiOx as HTL, (**ii**) NiOx as HTL with 0.8 wt% PTE, and (**iii**) NiOx as HTL with 1.5 wt% PTE and corresponding (**e**) histograms of grain size distribution for MAPbI_3_ with NiOx and NiOx with different solution concentrations (0.8 and 1.5 wt%) of PTE as HTL [81]. Reprinted with permission.

Heterogeneous nucleation is influenced by modifying the interfacial properties of the electron transport layer (ETL) or HTL on which surface perovskites are crystallized. Li et al. [80] introduced Cs_2_CO_3_ as a modifier to passivate defects at perovskite/SnO_2_ interfaces. They found that Cs_2_CO_3_ enhances surface wettability, which also aids in expanding the perovskite grain size, as demonstrated in the scanning electron microscopy (SEM) images in Figure 3a.

The device performance for Cs-doped SnO_2_ (Figure 3b) reveals that with the increasing concentration of Cs, the PCE is enhanced. All of the results acquired from approaches to modulating the surface energy demonstrate that the hydrophilic characteristic facilitates lowering the surface energy and encourages heterogeneous perovskite nucleation, producing high-quality perovskite films. Degani et al. [82] used the large organic cations 2-phenylethylammonium iodide (PEAI), 4-chloro-phenylethylammonium iodide (Cl-PEAI), and 4-fluoro-phenylethylammonium iodide (F-PEAI) to form both the top and bottom interfaces of PSCs with HTL/perovskite and perovskite/ETL interfaces. They reported that the bottom interface is more hydrophilic because of the polar nature of PEAI, which leads to the perovskite films being more compact and uniform without cracks and voids. They achieved a significantly improved interfacial wettability, which increased the PCE of the champion device to 23.7% for F-PEAI-modified devices. Liao et al. [83] pretreated NiO_x_ substrates with an aqueous solvent (H_2_O and N,N-dimethylformamide (DMF)), which increased the surface wettability and conductivity of the NiO_x_ layer during crystal growth, resulting in high crystallinity with large grains. The aqueous solvent accelerates the crystal growth in the preferred orientation. Liu et al. [84] prepared an interface buffer layer using an amphipathic conjugated molecule, betaine, for PeLEDs. They also showed that the betaine layers can control the grain size of the perovskite by increasing the number of crystal nucleation sites, resulting in an EQE of 11.1% with a smaller grain size. Tan et al. [85] introduced a bilateral interfacial chemical linker (2PACz), which can improve the crystallinity, the contact affinity between poly[bis(2,4,6-trimethylpheneyl)amine] and the perovskite layer, and the morphology of the perovskite film. The chemical linker can slightly change the crystallinity without changing the crystallographic structure of the perovskite films. The correlation between the grain size and contact angle for the one- and two-step spin-coating methods was discussed with the use of hydroxyl groups [81]. The hydrophilic surface due to the hydroxyl group in poly (ethylene glycol) tridecyl ether (PTE) on the underlying surface increased the grain size and established a positive correlation with device performance. They reported that expedited heterogeneous nucleation on the hydrophilic surface of a substrate is a supplement to the enhancement of grain size and higher crystallinity (Figure 3c,d). Nucleation and crystal growth are both present during the spin-coating process. Though at the initial stage of crystal growth, grains are dense with small sizes; because of some remaining DMF, grains become coarsened during thermal annealing.

## 3. Grain Size and Interfacial Crystallinity

**Figure 4:**
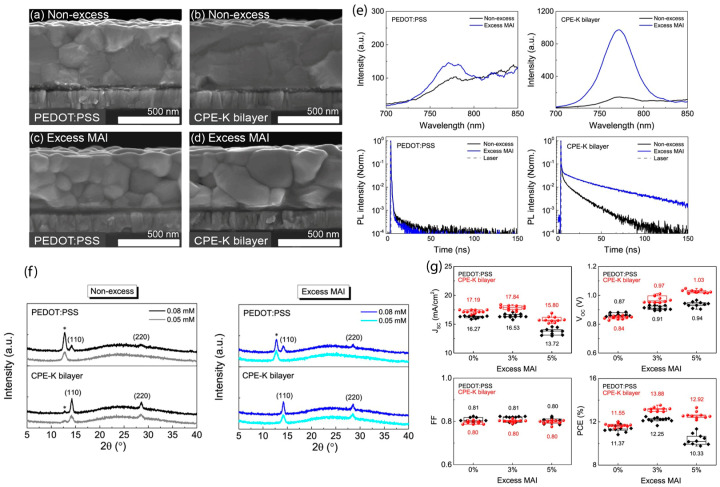
Interfacial crystallinity. (**a**–**d**) Top-view SEM images (scale bar 500 nm). (**e**) PL spectra and time-resolved PL spectra of perovskite films with PEDOT:PSS and CPE-K bilayer with excess and non-excess MAI. (**f**) XRD patterns of perovskite films formed from precursor solutions with different molar concentrations of PbI_2_ in DMF for non-excess (0%) and 5% excess MAI. The diffraction peak for the (001) plane of PbI_2_ is denoted by an asterisk. (**g**) Device performance for perovskite films with PEDOT:PSS and CPE-K bilayer with excess MAI [86]. Reprinted with permission.

The advantage of interfacial engineering can be found not only for wettability but also for the vertical growth of perovskites. Interfacial crystal quality is another key parameter that significantly influences the grain size and crystal growth. Kim et al. [86] established a direct relationship between the open-circuit voltage (*V*_oc_) and the interfacial properties of perovskites modified via stoichiometry engineering of the precursor solutions and the surface modification of the underlying PEDOT:PSS layer. They introduced a pH-neutral conjugated polyelectrolyte (CPE-K) at the interface between PEDOT:PSS and perovskites and reported that interfacial perovskite crystals can be improved by the synergetic effect of excess MAI and the CPE-K bilayer, which enhances *V*_oc_ by suppressing interfacial recombination. Moreover, they found that improved crystallization at the interface can accelerate the bulk growth of vertical perovskite grains and effectively suppress nonradiative surface charge recombination, thus enhancing the short-circuit current (*J*_sc_) and *V*_oc_. Figure 4 demonstrates that excess MAI utilizes the total PbI_2_ content for the crystallization of perovskite for the CPE-K bilayer. The corresponding device performance (Figure 4g) is also consistent with their statement. 

A tetradecylamine (TeDA) interfacial layer between NiO_x_ and a perovskite contact was introduced to study the effect of the interfacial layer on perovskite growth [87]. As an interlayer, TeDA can change the wettability of the NiOx film and simultaneously enhance the charge extraction efficiency. The increase in the contact angle indicates that TeDA modifies the surface energy of the NiOx surface, which results in a more hydrophobic surface of the NiOx film. The grains on the TeDA-treated NiOx films were found to be larger than those on the films without TeDA. With a hydrophobic surface, heterogeneous nucleation is obstructed, which leads to less dense nuclei and, consequently, a larger grain size. XRD analysis of the perovskite with TeDA-treated NiO_x_ films showed a marked increase in the intensity ratio of the (100) and (200) peaks, indicating higher crystallinity with larger grains. Xu et al. [88] studied the CsI-SnO_2_ interlayer and showed that the perovskites on the CsI-SnO_2_ complex grow more vertically aligned to the substrates in terms of grain morphology, with a lower grain boundary area for charge carriers to use to transport through the layer. A multifunctional interfacial material (biguanide hydrochloride (BGCI)) [89] was used as the SnO_2_/perovskite interlayer. The certified PCE was considerably increased to 24.4% with a *V*_oc_ of 1.19 V, which suggests that BGCI, when used as an interfacial material, effectively modifies the interfacial crystallinity with controlled crystallization in SnO_2_/perovskite. They found that PbI_2_ and perovskites that crystallized on the BGCI interfacial layer had increased crystal sizes, with the homogeneous crystal growth of PbI_2_ and the formation of a uniform perovskite film with large grains. Tong et al. [90] demonstrated multifunctional interfacial engineering using KMnO_4_ and examined the diffusion of K^+^ ions into perovskite films with larger grains. Further, they found that the Mn^2+^ ions improved both the crystallinity and phase stability of the perovskite film. K^+^ and Mn^2+^ diffuse from the surface layer of ETL (SnO_2_) into the bulk of perovskite, which increases the grain size with high crystallinity.

## 4. Polymer-Assisted Crystallization

**Figure 5:**
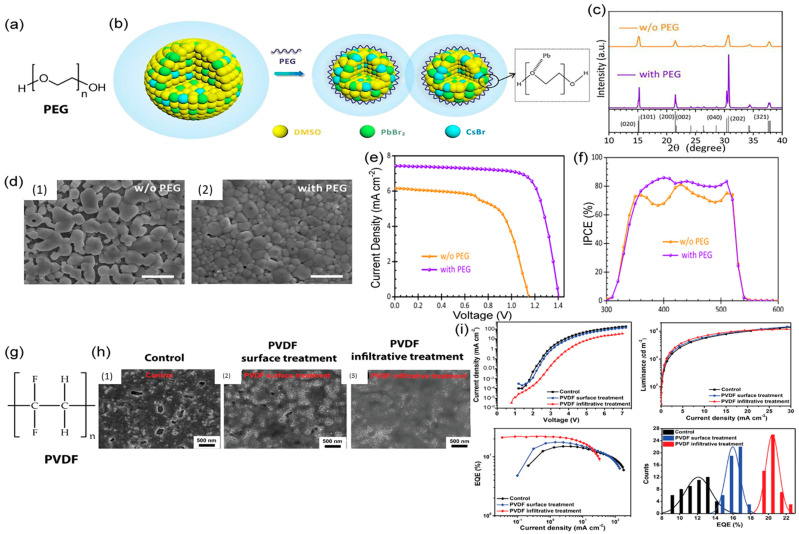
Polymer-assisted crystallization: (**a**) molecular structure of polyethylene glycol (PEG); (**b**) an illustration of the increase in the number of colloids with the aid of PEG; (**c**) X-ray diffraction (XRD); (**d**) top-view (SEM) images of calcined CsPbBr_3_ perovskite thin layers prepared with and without PEG (the scale bar is 1 µm); (**e**) *J−V* curve and (**f**) IPCE (%) vs. wavelength of corresponding CsPbBr_3_ films [92]; (**g**) molecular structure of poly(vinylidene fluoride) (PVDF); (**h**) top-view SEM images for control, surface, and infiltrative treatment with PVDF (scale bar is 500 nm); and (**i**) device performance of corresponding PeLEDs [97]. Reprinted with permission.

Polymer-assisted crystallization is another intriguing approach widely utilized to realize high-quality crystals by modulating the nucleation and growth kinetics. Yan et al. [91] used a polymer-controlled nucleation technique to synthesize large-size mixed-cation and mixed-halide perovskite single crystals. They investigated different polymer ligands, such as polyethylene glycol (PEG), polypropylene glycol (PPG), polyacrylic acid (PAA), and polyvinyl alcohol (PVA), that contain oxygen groups, and with the aid of these polymer ligands, the nucleation process can be controlled, and large-size high-quality organic–inorganic perovskite single crystals are formed at a rapid growth rate. The method affords a suitable coordination interaction between the polymer oxygen groups and Pb^2+^, which results in a four-fold reduction in the nuclei concentration. The nuclei concentration is associated with the supersaturation state. The fast growth caused by high supersaturation is usually followed by the rapid formation of a significant number of nuclei, resulting in a massive number of small single crystals. High supersaturation may also degrade the stability of the growth solution, allowing impurity phases and defect structures to form. The oxygen groups of these polymers occupy some of the coordinating Pb (II) ions, substituting coordinating iodide ions and solvent molecules in the precursor solution. The Pb-I_6_ complex is formed in the supersaturation state, and it serves as a nucleus that drives the formation of centimeter-sized single crystals; thus, the number of nuclei is reduced. Another study [92] incorporated PEG as a polymer additive into the precursor solution of an all-inorganic CsPbBr_3_ perovskite material to boost the number of clusters of CsBr-PbBr_2_-DMSO-PEG (DMSO: dimethyl sulfoxide) colloids. Because of the lower nucleation free-energy barrier, these colloids acted as a support and promoted heterogeneous nucleation on the surface, by which the surface coverage of the perovskite film was significantly improved. The results shown in Figure 5d indicate that the PEG-treated perovskite films led to the formation of more compact and dense grains after annealing. The device performance (Figure 5e) and IPCE (Figure 5f) also indicate that with PEG, the photovoltaic parameters are significantly enhanced. The perovskite film’s surface coverage is greatly enhanced by enabling rapid nucleation. Figure 5c shows proof of higher crystallinity with PEG-assisted perovskite films. Pyridine was introduced [93] to control the rapid crystallization of tin-based perovskites. As DMSO induces a slow crystallization rate, it oxidizes tin while processing perovskite films. Therefore, DMSO was replaced by pyridine, which does not oxidize tin during controlled crystallization. The pyridine treatment effectively controls nucleation and crystal growth and results in films with pinhole-free morphologies and large grains. Zhao et al. [94] proposed an improved solution process for tandem-like perovskite photodetectors using poly(methyl methacrylate) (PMMA) as an antisolvent. PMMA promotes the growth of perovskite crystals with large grains and reduces the horizontal grain boundaries. The carbonyl group that was developed as an intermediate adduct in PMMA and PbI_2_ promotes nucleation with slower crystal growth, which results in highly crystalline perovskite films. The authors reported that the photodetector exhibited a peak-specific detectivity of 3.38 × 10^12^ Jones with a high responsivity (5.65 A/W) and EQE (1321%) under 532 nm illumination and a small bias of −1 V. 

Three-dimensional (3D) polymers having multiple branches and functional groups have been utilized. A 3D star-shaped polymer, polyhedral oligomeric silsesquioxane-poly(trifluoroethyl methacrylate)-*b*-poly(methyl methacrylate) (PPP) [95], which serves as a modifier for perovskite crystallization, was reported. The integration of several chemical anchor sites in the star-shaped polymer branches significantly influences the crystallization of perovskite films with a reduced trap density and higher carrier mobility, inhibiting nonradiative recombination and lowering charge-transport loss. The PPP polymer branches have carbonyl (C=O) and -CF_3_ that act as chemical anchor sites. They effectively suppress nonradiative recombination by defect passivation and also increase the stability of the perovskite film under different environmental conditions of moisture, thermal, or illumination stress. The PPP polymer is applied through antisolvent engineering with different solution concentrations. Therefore, the PPP-regulated PSC exhibits an optimal PCE of 22.1% with a high fill factor (0.862). Another polymer, polyvinyl butyral (PVB), has been studied and used in a broad range of applications in the field of optoelectronics because of its high optical quality, surface adhesion properties, and resistance to water and heat. PVB [96] was used as a polymer additive to develop PSCs with better crystallinities, larger grains, and fewer defects. Compared to the control device, the PVB-modified PSC exhibited a noticeable PCE improvement from 16.34% to 19.04% with a higher *J*_sc_ (~23 mAcm^−2^), *V*_oc_ (1.06 V), and fill factor (77%).

Polymer-assisted crystallization has been applied to realize high-performance PeLEDs via a reduction in bulk or surface defects by fine-tuning perovskite nucleation and crystal growth. Feng et al. [97] developed a polymer infiltrative treatment method, in which polymers are blended as antisolvents into the perovskite films before their complete crystallization. The authors observed that the antisolvent-assisted modulation of the crystallization process without PVDF resulted in heterogeneous nucleation with uncontrolled crystal growth. By contrast, when the semi-crystalline perovskite films were infused with the PVDF solution, the PVDF polymer chains blended into the perovskite matrix. The F atoms in the PVDF polymer chains chemically interact with the perovskite and slow the grain growth, adding to the dual effects of defect passivation and controlled crystal growth. These high-quality green PeLEDs showed a maximum EQE of 22.29% (Figure 5i) with void-free surfaces and few-defect crystals, as evident from SEM images (Figure 5h). A polymeric interlayer (polyvinyl pyrrolidone, PVP) [98] was incorporated into cesium-based PeLEDs. The EQEs of these PVP-assisted PeLEDs were four-fold higher than those of the control device. The fabricated devices revealed a dense crystal distribution on the PVP layer, demonstrating that perovskite precursor nucleation on the PVP substrate was faster than without the PVP layer owing to its lower free-energy barrier for heterogeneous nucleation. Further, FTIR spectra of the devices indicated the formation of the [C=O–Pb^2+^] complex during the spin coating of the perovskite precursor on the top of the PVP layer.

## 5. Perovskite Crystallization with Solution Properties

**Figure 6:**
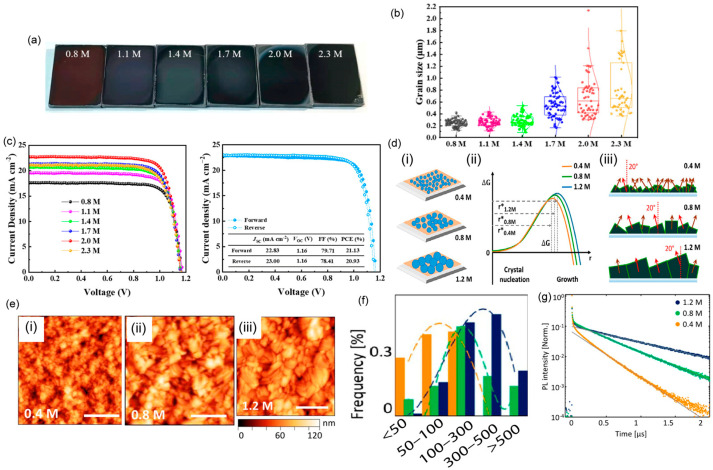
Effect of precursor concentration. (**a**) Photographs and (**b**) grain size distribution of perovskite films with different precursor concentrations. (**c**) *J−V* curve for different precursor concentrations and forward and reverse scans of *J−V* curve for champion device for triple-cation perovskite films [99]. (**d**) (**i**) Schematic of grain growth, (**ii**) nucleation and Gibbs free energy relation with different concentrations as a function of critical radius, r*, and (**iii**) effect of grain orientation influenced by grain growth process for different precursor concentrations of triple-cation perovskite films. (**e**) AFM images (the scale bar is 1 µm) of the films prepared with different concentrations; (**i**) 0.4, (**ii**) 0.8, and (**iii**) 1.2 M of the mixed-ion perovskite. (**f**) Grain size distribution for different concentrations of precursor solution (0.4, 0.8 and 1.2 M) and (**g**) time-resolved PL for different precursor concentrations [102]. Reprinted with permission.

Perovskite crystallization can be affected by changing the parameters of the precursor solutions used for synthesizing perovskites, including the solution concentration, solvent effect, and antisolvent influence. Not only the film thickness but also the crystallinity and morphology of perovskite films depends on the precursor-solution concentration, which can influence the trap states in the films. With increasing precursor concentration, the grain size increases, which results in reduced grain boundaries and fewer trap states, which therefore leads to high-performance perovskite films [99,100,101]. Du et al. [99] reported that an increase in the precursor concentration accelerates grain growth, reducing grain boundary and bulk defects. They demonstrated SEM images for different precursor concentrations, showing that with increasing concentration, the grain size increases (Figure 6b). They emphasized that with increasing concentration, as the number of nuclei is assumed to be constant, only the grain size is increased with higher supersaturation during the annealing stage. As a result, their device efficiency increased to ~21% (Figure 6c), which was a significant improvement over that of the pristine device. Figure 6a presents photographs of the perovskite films prepared with different precursor concentrations. As the concentration increases, the films become denser and darker relative to those prepared using low concentrations of the precursor solution. 

Wieghold et al. [102] investigated the influence of the precursor concentration on mixed-halide perovskite thin films. They revealed that grains in the polycrystalline thin films grow larger and more faceted with increasing precursor concentration. The films with large grains were oriented at an angle of 20° relative to the surface normal on the (100) plane, whereas those with small grains primarily displayed random grain orientation angles. Figure 6d depicts the schematic illustration of grain growth and presents the relation between nucleation and Gibbs free energy with varying precursor concentrations. AFM images and histograms of the grain size distribution (Figure 6e–g) clearly reveal that with changing precursor concentrations, the crystallinity and crystal growth are greatly affected. Abnormal grain growth was demonstrated [103] on a TiO_2_ film, and the interfacial properties with changing concentrations of PbI_2_ were investigated. With increasing solution concentration (up to 1.2 M), the average grain size increased, and the interfacial crystallinity of perovskite films improved. Ezike et al. [100] conducted micro-structural analyses and reported an increase in the XRD peak intensity with increasing precursor concentration, indicating high crystallinity and large grain sizes. They reported that with the optimal precursor concentration, surface recombination can be suppressed in an effective way, which reduces trap-assisted recombination and leads to high-performance PSCs. Moreover, light absorption is increased with higher precursor concentrations, and the film thickness increases as well. They found that the perovskite bandgap was reduced with increasing precursor concentration because of the formation of sub-bands due to defect-associated forbidden gaps of MAPbI_3_. In addition to the aforementioned studies, several researchers [101,104,105,106] have demonstrated the modification of the grain size and crystallinity in perovskite devices by varying the precursor concentration. It was demonstrated that by changing the molar ratio of PbCl_2_ and CH_3_NH_3_PbI_3_ with varying molar concentrations of PbCl_2_, a pinhole-free perovskite film with a suitable energy bandgap and higher optical absorption can be acquired. PbCl_2_ colloids were considered heterogeneous nucleation sites that increase with the addition of excess PbCl_2_, which improved the morphology of the perovskite film [105]. 

The process by which supersaturation is achieved influences several crystal properties, including the size, morphology, and coexisting atomic configurations of perovskite materials. Gas flow and antisolvent crystallization are approaches to intentionally reaching the supersaturation state. The gas-flow method was used to accelerate the evaporation of the solvent, which leads to fast nucleation to form high-quality perovskite films [107,108]. This gas-flow method was employed instead of an antisolvent to eliminate non-complexed solvent and thus eliminate the impact of solvent–solvent interactions in the liquid precursor phase, resulting in the establishment of a supersaturated intermediate state and thus efficient n-i-p-type solar cells with PCEs of more than 20% [109]. Gas-flow approaches have been found to be more promising for the up-scaled production of perovskite films in practical applications than the antisolvent technique, which has traditionally been utilized to fabricate perovskite films for research purposes. It not only avoids the use of hazardous antisolvents but also produces high-quality perovskite films with great repeatability and resilience. More crucially, because of the adjustable supersaturation, gas quenching allows for more exact control of the crystallization process. As a result, the air-blade treatment based on the gas-quenching mechanism has grown in popularity in the fabrication of large-area perovskite films and modules [110,111].

**Figure 7:**
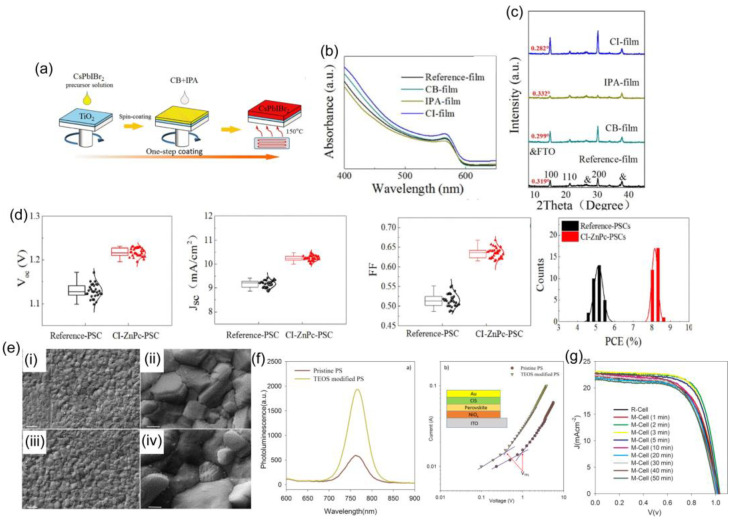
Antisolvent treatment. (**a**) Schematic illustration of one-step spin-coating process with modified antisolvent treatment. (**b**,**c**) XRD and UV–vis absorption spectra of pristine and CB-, IPA-, and CI-treated films deposited on the glass/FTO/TiO_2_ substrate. (**d**) Device performance corresponding to zinc phthalocyanine (ZnPc)-modified HTL with mixed antisolvent (CB+IPA)-treated films [119]. (**e**) Top-view SEM images with pristine (**i**,**ii**) and TES-modified perovskite films (**iii**,**iv**). Scale bars are 1 µm (**i**,**iii**) and 200 nm (**ii**,**iv**). (**f**) Steady-state PL spectra of pristine Cs_0.05_ (MA_0.17_ FA_0.83_)_0.95_ Pb (I_0.83_ Br_0.17_)_3_ PS and TEOS-modified PS films and current–voltage properties of hole-only devices with ITO/NiO*_x_*/perovskite/CIS/Au structure (inset of the figure). (**g**) *J−V* curve for reference and TES-modified devices with different time intervals after TES addition [118]. Reprinted with permission.

Due to the slow nucleation rate and early crystallization of perovskite materials, which result in the formation of needle-like structures, the spin-coating process typically results in insufficient surface coverage. This limitation can be overcome by using an antisolvent (such as toluene or chlorobenzene) during the initial spin coating of the DMF-added PbI_2_ and MAI solution [112,113]. Although gas flow was applied in previous studies to increase the crystallization rate, current studies focus on antisolvent-based one- and two-step spin-coating methods because of their simplicity and cost-effectiveness. There are numerous beneficial aspects for antisolvent crystallization, such as a low operating temperatures and interaction with the solvent, which affects the polymorphic form of the perovskite for final crystal growth. An antisolvent is typically added to the precursor solution at some point while spin coating. The antisolvent extracts the precursor solvent during the spin-coating process, and as a result, the solution rapidly reaches the supersaturation state. Thus, antisolvent incorporation accelerates the nucleation, resulting in the formation of more nuclei on the substrate surface. As the concentration drops below the threshold level for nucleation, a growth phase occurs, and tightly packed films with complete substrate coverage are produced. The resulting films are then thermally annealed to remove the remaining solvent [114,115,116,117,118,119]. Yang et al. [119] reported an antisolvent technique with chlorobenzene (CB) and isopropyl alcohol (IPA). Figure 7b depicts the UV-vis absorption spectra of the matched CsPbIBr_2_ films. The CI-film has the best absorption, which helps to improve the short-circuit current density (*J*sc).

Figure 7c presents the X-ray diffraction (XRD) patterns of CsPbIBr_2_ films deposited on FTO/TiO_2_ with various antisolvents. In comparison to the other films, the CI-film has the highest peak intensity and the lowest FWHM value. They recommended the optimal crystallinity and orientation of CsPbIBr_2_ grains in the CI-film. All of the CsPbIBr_2_ films have the same absorption edge of ~599 nm, which corresponds to a bandgap of 2.07 eV. The device performance (Figure 7d) for zinc phthalocyanine (ZnPc)-modified HTL exhibits higher performance than pristine devices with mixed antisolvents. Samadpour et al. [118] introduced an antisolvent dripping method that incorporates tetraethylorthosilicate (TEOS). Top-view SEM images correspond to denser and larger grains than those of the pristine film (Figure 7e). Figure 7f shows the PL spectra for pristine and TEOS-modified PSCs, and the PL intensity was markedly enhanced after modification. Additionally, the density of trap states was measured with SCLC measurements (Figure 7g), and the trap density was significantly reduced after treatment with TEOS. Therefore, the antisolvent and thermal annealing both play a key role in inducing fast crystallization on perovskite films.

The solvents for perovskite solutions also affect nuclei or growth, including by slowing reaction rates and coarsening grains for the formation of perovskite films [120]. Typically, solvents such as DMF, DMSO, N-methylpyrrolidone (NMP), and gamma-butyrolactone (GBL) are used to prepare perovskite solutions. If the nucleation/growth process can be controlled during PbI_2_ crystallization, it facilitates the formation of a mesoporous film via two-step spin coating. Because of the weak interactions between DMF and Pb^2+^ (D_N_(DMF) = 26.6 kcal mol^−1^), the low boiling point of DMF (152 °C), and its fast evaporation, PbI_2_ is quickly crystallized, leading to a non-uniform surface coverage [121]. Many investigations [122,123,124,125] on MAPbI_3_ perovskite films reveal that a DMF-DMSO binary solution can slow the crystallization rate by synthesizing MAI-PbI_2_-DMSO complexes and lowering the nucleation rate of MHPs, thus leading to higher quality films.

## 6. Heterogeneous Nucleation of Quasi-2D Perovskites

**Figure 8:**
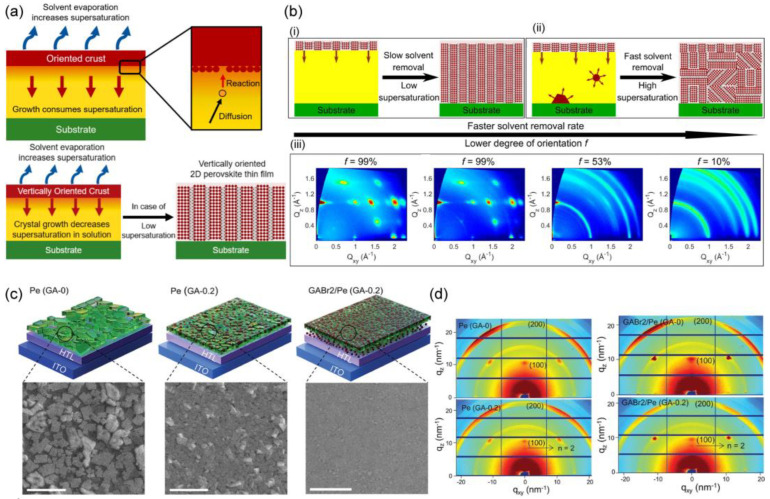
Heterogeneous nucleation of quasi-2D perovskites. (**a**) Schematic diagram of supersaturation process indicating fast solvent evaporation and formation of vertically oriented 2D perovskite. (**b**) (**i**) At lower supersaturation, continuity of crystal growth with slow removal rate of solvent of vertically oriented 2D perovskite (not to scale); (**ii**) introduction of orientation randomness with fast solvent evaporation; (**iii**) GIWAXS patterns with increasing solvent removal rate, including slow, medium, fast, and fastest, accordingly [126]. (**c**) Schematic illustration of doping effect of GABr on HTL with corresponding SEM images (scale bars are 500 nm). (**d**) GIWAXS patterns for different doping concentrations of GABr show different peak positions (red color) along the *q*_z_ axis, indicating that the layered perovskites are aligned horizontally to the substrate [128]. Reprinted with permission.

Quasi-2D perovskite films for photovoltaics have been intensively investigated, as the hydrophobicity of large organic cations slicing the 3D perovskite framework enhances the air stability of films and devices. Sun et al. [43] used L-Norvaline to develop a COO-coordinated intermediate phase with a low formation enthalpy. This new intermediate phase suppressed phase segregation, thereby modifying the crystallization process. As a result, high-quality large-area quasi-2D films were produced. In recent years, anion engineering has emerged as a successful method for modulating intermediate phases and controlling crystallization. The strong binding affinity of the COO- anion, as a bidentate ligand, with the perovskite precursor results in the formation of the intermediate phase. The orientation of quasi-2D perovskites is determined by the competition of heterogeneous nucleation at the air interface over homogeneous nucleation. Chen et al. [126] presented a general mechanism for the crystallization of 2D perovskites, in which the competition between solvent evaporation and crystal growth noticeably influences the degree of supersaturation, and low supersaturation is required to crystallize vertically oriented thin films, beginning with nucleation at the liquid–air interface (Figure 8a). The degree of crystallographic orientation is strongly influenced by diverse factors, such as organic spacers, solvents, and solvent evaporation rates. Nucleation is favored in the context of high supersaturation, resulting in additional nucleation. These extra nuclei can occur within the bulk liquid phase as well as at the substrate–liquid interface, as seen in Figure 8(bii). Because of the van der Waals interactions between 2D crystal plates and the substrate, the liquid–substrate contact often produces a horizontal orientation. Figure 8(biii) shows the GIWAXS characterization of the degree of crystallographic orientation under various solvent removal conditions. For the film obtained by the slow evaporation of the solvent, the grains are highly oriented.

Li et al. [127] proposed and employed mixed pseudo-halide anion engineering to synthesize quasi-2D RuddlesdenPopper perovskites, with a target formula of PEA_2_FA_4_Sn_5_I_16_, using acetate (Ac^−^) and tetrafluoroborate (BF_4_^−^) anions. The mixed Ac^−^ and BF_4_^−^ anions can effectively break PEA^+^-PEA^+^ stacking in the precursor and result in a uniform distribution of PEA^+^ cations. However, they can also delay crystallization by coordinating with the unbonded SnI_2_ and thus significantly reduce the fraction of low-*n* 2D phases, enhance the crystal orientation, and suppress Sn^2+^ oxidation. The observed increase in the grain size of the PEABF_4_ thin film showed that BF_4_^−^ can slow the crystal growth and nucleation of 2D and quasi-2D phases. Guanidinium bromide (GABr)-based quasi-2D PeLEDs were introduced [128], wherein the GA^+^ ions acted as spacer cations (Figure 8c). As with the XRD findings, there is a rise in diffraction intensities (Figure 8d) when examining the perovskite films grown on the pre-deposited GABr layer. The GA^+^ ions were introduced into both the perovskite precursor solution and the interlayer between the HTL and the perovskite. The interlayer provided more nucleation sites, whereas the added GA^+^ cations in the precursor reduced the crystallization rate by forming strong hydrogen bonds with the intermediates. As a result, the authors could successfully fabricate quasi-2D phase PeLEDs with an EQE of more than 20%, afforded by the spacer cation strategy.

Lee et al. [129] introduced a quick and comprehensive stamping technique to fabricate reverse-graded, highly aligned perovskite crystals with low-*n*-value 2D perovskite phases on the film surfaces. The contact interface between the 2D and 3D films initiated heterogeneous nucleation during the stamping process, resulting in well-crystallized perovskite films aligned out-of-plane with respect to the substrate. They added that while stamping, the evaporation rate of solvents used for the precursor became slow. This suppressed evaporation ensures heterogeneous nucleation with the downward growth of a 2D perovskite from the contact substrate, leading to the favorable orientation of perovskite crystals for photovoltaics. Thus, a highly oriented and reverse-graded quasi-2D perovskite with an average *n* value of 18 was produced. The corresponding *iso*-butylammonium (*iso*-BA)-based (*iso*-BA_2_MA*_n_*_−1_Pb*_n_*I_3*n*+1_) PSCs exhibited a high *V*_oc_ of 1.11 V and a PCE of more than 17%. Potassium bromide [130] is applied as an additive that can cause multiphase perovskite grains to self-assemble into a bilayer microstructure. Such a structure is observed because the potassium bromide additive can create heterogeneous nucleation templates for the crystallization of 3D perovskite grains on the precursor–substrate bottom interface. Their facilitated nucleation can be described in such a way that KBr phases are precipitated first on the solution–substrate interface, which might act as a nucleation template for the larger grain growth of a 3D perovskite. Additionally, KBr dispersed in the precursor solution promotes heterogeneous nucleation within the bulk of the perovskite film. Using this bilayer film microstructure, the authors could significantly increase the EQE of quasi-2D PeLEDs. A series of alkali-bromides [131] were utilized to regulate the nucleation and crystal growth of quasi-2D PeLEDs. The authors reported that the addition of K^+^ prevents the nucleation of high-*n* phases and promotes the growth of low-*n* phases, resulting in a uniform spatial distribution of different n phases and improved energy transfer. This method can be used to fabricate efficient green quasi-2D PeLEDs with a champion EQE of 18.15% and a maximum brightness of 25,800 cd m^−2^. Overall, the growth retardation of low-n phases is an effective technique to produce Li-, Na-, and K-added films with enhanced crystallinities and highly ordered crystal structures.

## 7. Lattice Mismatch and Lattice Constant

The lattice constant is also an important parameter when introducing interlayers into perovskites. Not only the number of nuclei but also the lattice constant can be changed by external doping, which severely impacts the performance of perovskite films. External doping can lead to the mismatch of crystal lattice constants, resulting in a certain distortion of the degree of crystallinity [132]. Yang et al. [133] demonstrated that with a higher volume ratio of K^+^ doping (~6%), the photovoltaic performance is degraded because of a mismatch in lattice crystallinity. Therefore, they chose the optimal volume ratio (~3%) that provided the best photovoltaic performance. To eliminate the interfacial lattice mismatch, Zhang et al. [134] adopted a p-chlorobenzenesulfonic acid (CBSA) self-assembled small molecule (SASM) to perform dual passivation for NiO_x_ and perovskite crystals. Monofunctional molecules (CB, BSA) exert an excellent passivation effect either on perovskite or on NiO*_x_*, where CBSA can both achieve dual passivation and act as the growth inducement platform for subsequent perovskite growth with the elimination of the interfacial lattice mismatch.

For quasi-2D perovskites, Kepenkian et al. [135] revealed that perovskite layers over a threshold thickness cause the interfacial strain to relax, releasing the mechanical energy created by the lattice mismatch, which sparks surface reorganization and might cause the emergence of the previously noted lower energy edge states. It is believed that these states, which are absent in three-dimensional perovskites, will be very crucial in the development of lead-halide perovskites for optoelectronic systems. Qin et al. [136] described a simple method using perovskite quantum dots (PQDs) to achieve the epitaxial-like development of highly oriented tin-based perovskite films. PQDs can serve as nucleation centers to drive the formation of highly oriented perovskite crystals for both FASnI_3_ and MASnI_3_ systems. Notably, by adjusting the lattice mismatch between different PQDs and bulk perovskites, the amount of lattice strain may be easily controlled to reduce the defect density and increase the efficiency with the matching lattice constant. According to this study, MASnI_3_ PSCs with PQDs have the highest documented efficiency of this type (12.49%).

## 8. Effect of Heterogeneous Nucleation on Single-Crystal and Nanocrystal Perovskites

Single-crystal perovskites exhibit a promising increase in optoelectronic performance because they are free of grain boundaries, while in polycrystalline perovskites possessing grains, ion migration, surface defects, and chemical instability cause degradation in the optoelectronic performance of MHPs. There are different growth mechanisms for single-crystal perovskites, including the inverse-temperature crystallization method, the solvent-evaporation crystallization method, and the antisolvent-vapor-assisted crystallization method [137,138].

The fabrication of perovskite single-crystal thin films in a controlled way is a challenging task in developing high-performance optoelectronic applications. The substrate surface energy modification has been used to control the growth of single-crystal thin films on substrates via the space-confined method. Di et al. [139] demonstrated that the hydrophobicity of ITO substrates, which were modulated by UV–ozone treatments, suppressed heterogeneous nucleation, enabling slow but continual growth for the formation of single crystals with a large grain size from few nucleation sites. Likewise, Pratheek et al. [140] also reported a large-area ultrathin wafer of MAPbBr_3_ via a diffusion-facilitated inverse-temperature crystallization (DFITC) method by applying a hydrophobic hole transport layer (PTAA) on ITO substrates. Without PTAA on the ITO substrates, a large number of small crystals were formed, whereas with PTAA, the number of crystals was reduced, but the crystal sizes became larger. Deng et al. [141] also produced a centimeter-scale single-crystal perovskite thin film on a hydrophobic substrate via the spatially confined growth method. They also observed that with the hydrophobic nature of the substrate, the nucleation on the substrate surface was retarded, whereas the growth of single crystals was enhanced. Chen et al. [142] reported that when substrates were coated with hydrophobic PTAA, the diffusion rate of ions was significantly increased. This enhanced ion diffusion rate in micrometer-scale gaps allowed for the continuous growth of thin single-crystal perovskite structures that can extend up to several millimeters along the in-plane direction.

Additive engineering or seed-induced crystal growth has added new approaches to heterogeneous nucleation-dependent single-crystal growth. Xiao et al. [143] introduced a new chemical, SnF_2_, which was used as an additive in lead-free tin-based perovskite solar cells and can accelerate heterogeneous nucleation by forming both thin films and single-crystal perovskites. The solubility of SnF_2_ is limited, which enables more nuclei that ensure the full coverage of the thin film. The nuclei act as heterogeneous nucleation centers to accelerate the growth of MASnIBr_2_ crystals with the uniform coverage of the thin film. They observed that with 30 mol% excess SnF_2_, the grain size became twice that obtained without SnF_2_. Two-inch-sized crystals of MAPbX_3_ (where X = Cl^−^, Br^−^, I^−^) were obtained by using a seed-induced heterogeneous nucleation mechanism [144]. The seed crystal acts as a heterogeneous nucleation site where additional crystal layers can grow more rapidly than in the surrounding solution. This is because the surface of the seed crystal provides a favorable orientation for crystal growth, allowing the crystal lattice structure of the surrounding solution to match more easily and efficiently. The use of seed-induced nucleation can be important in controlling the size and morphology of crystals for various applications. To initiate the space-confined solvent-evaporation-induced crystallization process, an array of perovskite seeds was pressed onto a substrate [145]. In order to create the stamped seed array, perovskite inks were first printed onto a temporary substrate, which resulted in the formation of ordered perovskite seeds as the ink droplets evaporated. The stamp with the perovskite seed array was then transferred onto a target substrate containing a saturated precursor solution. The stamped seed array facilitated heterogeneous nucleation while effectively impeding random homogeneous nucleation. As the solvent gradually evaporates at room temperature [145], single-crystal thin films with patterned geometries can be grown on the uniformly distributed seeds on the entire substrate. This technique enables the scalable growth of millimeter-sized single-crystal halide perovskite thin-film arrays with controllable thicknesses ranging from hundreds of nanometers to over 10 μm.

**Figure 9:**
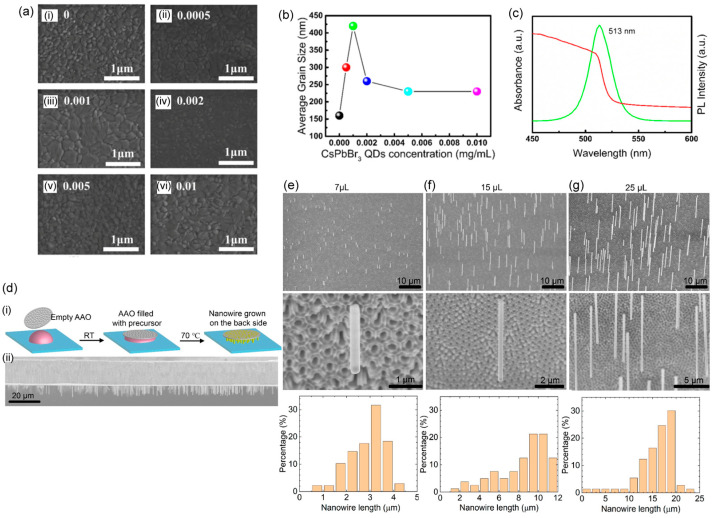
Heterogeneous nucleation effect on nanocrystals. (**a**) Top-view SEM images of different concentrations ((**i**) pristine; (**ii**) 0.0005 mg mL^−1^ (**iii**) 0.001 mg mL^−1^ (**iv**) 0.002 mg mL^−1^ (**v**) 0.005 mg mL^−1^ (**vi**) 0.01 mg mL^−1^ of CsPbBr_3_ QDs in diethyl ether, with scale bar 1 µm. (**b**) Average grain size vs. different solution concentrations of CsPbBr_3_ QDs from 0 to 0.01 mg mL^−1^. (**c**) UV-vis absorption (red) and steady-state PL spectra (green) for CsPbBr_3_ QDs [148]. (**d**) Illustration of growth process (**i**) and cross-sectional SEM images (**ii**); scale bar is 20 µm of free-standing CsPbBr_3_ nanowires with back side of AAO template. SEM images and statistical distribution of the length of the free-standing nanowire arrays, grown by using (**e**) 7 µL, (**f**) 15 µL, and (**g**) 25 µL of precursor solution. The sample stage is tilted by 30° [149]. Reprinted with permission.

The crystal growth of perovskite nanocrystals can be affected by heterogeneous nucleation. The MHP nanocrystal includes zero-dimensional quantum dots (QDs) and one-dimensional nanorods or nanowires. CH_3_NH_3_PbBr_3_ quantum dots serve as nucleation centers when the QD solution in an antisolvent was used for spinning the perovskite precursor to realize perovskite-based light-emitting solar cells (LESCs) [146]. The devices exhibited an external quantum efficiency (EQE) one magnitude higher than that of the control device. Simultaneously, the photovoltaic PCE was increased from ~14 to ~17%. Inorganic halide perovskite QDs (IPQDs) require the minimization of the size distribution and the scaling of the synthesis process. To overcome these issues, a one-pot method was introduced via heterogeneous nucleation agents that occur on the surfaces of inert chemicals, which impedes the accumulative repeated growth of primary IPQDs and allow homogeneous nucleation to form diverse IPQDs [147]. Yao et al. [148] investigated CsPbBr_3_ QDs as an additive in an antisolvent for solution-processed MAPbI_3_ perovskite films. The CsPbBr_3_ QDs act as heterogeneous nucleation centers, which results in pinhole-free high-quality perovskite films with larger grain sizes. The role of CsPbBr_3_ QDs can be explained by the fact that the perovskites prefer to grow surrounding the QDs, which eases the growth of perovskite crystals with larger grain sizes. Figure 9a shows that with increasing CsPbBr_3_ concentration in the antisolvent, the film became denser with larger grains. With excessive heterogeneous seeds of CsPbBr_3_ QDs, the grain size became smaller (Figure 9b).

It was demonstrated that CsPbBr_3_ perovskite nanowires can significantly impact the surface electronic properties of MAPbI_3_ perovskite thin films [150]. The nanowires can be effective sites for heterogeneous nucleation, promoting the crystallization of the perovskite–solvent adduct thin films. In addition, nanowires were utilized to decrease the surface roughness of the MAPbI_3_ films, which is attributed to the availability of additional heterogeneous nucleation sites that can accelerate the nucleation rate during the MAPbI_3_ crystallization process. Zhang et al. [149] employed a low-temperature solution-based technique for synthesizing self-standing vertical CsPbBr_3_ nanowires that emit green light. These nanowires were grown on anodized aluminum oxide templates (AAOs) (Figure 9d). The adequate surface area of the nanopore AAO template for heterogeneous nucleation allowed the nucleation of nanowires to occur inside the nanopores. The length of the nanowires was controlled by varying the amounts of precursor solutions used for their growth. SEM images of nanowires (Figure 9e–g) grown using different precursor solutions show that all of the nanowires are aligned vertically and have a uniform growth direction. Spina and coworkers [151] discussed the growth of photovoltaic CH_3_NH_3_PbI_3_ nanowires using graphoepitaxial liquid–solid growth. They used this regulated liquid–solid growth approach to create a mm^2^-sized surfaces made of nanowires. According to their findings, individual nanowires were developed on narrower channels, but wider channels were filled with groups of nanowires. The randomized character of heterogeneous nucleation was employed to explain these events. Since narrower channels were smaller than the size of the first nucleus produced, it was expected that they would have fewer nucleation centers, resulting in prolonged single crystallites. Recently, nitrogen-doped carbon nanofibers were utilized to guide the crystal orientation of stable perovskite solar cells by regulating heterogenous nucleation, which resulted in a substantial increase in the power conversion efficiency (PCE) of the cells from 21% to 24% [152]. The photovoltaic enhancement was explained by the interaction between the nitrogen-based functional groups and PbI_2_, creating more heterogeneous nucleation sites with a preferential crystal orientation.

## 9. Temperature Dependence of Nucleation and Crystal Growth

**Figure 10:**
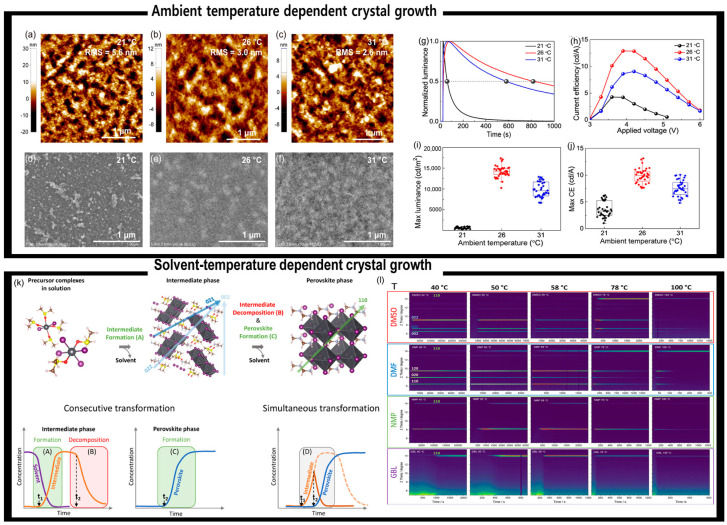
Temperature-dependent crystal growth. Temperature-dependent crystal growth: AFM (top) and SEM images (bottom) (scale bar is 1 µm) of the (PEA)_2_MAn–1Pb_n_Br_3n+1_ layered perovskite films spin-coated at an ambient temperature of (**a**,**d**) 21 °C, (**b**,**e**) 26 °C, and (**c**,**f**) 31 °C and (**g**–**j**) device performance for of perovskite films with different ambient temperatures [153]. (**k**) Schematic illustration of different processes, from perovskite crystal growth to formation of perovskite film (top) and consecutive and simultaneous transformation represented via kinetic curve (bottom). (**l**) Two-dimensional contour plots of GIWAXS detector frames in relation to time frame during crystallization of MAPbI_3_ from DMSO, DMF, NMP, and GBL at different temperatures [154]. Reprinted with permission.

We have discussed the fact that the evaporation rate of the solvent is a key parameter determining the nucleation kinetics. The temperature cannot be overlooked as a crucial parameter that determines the nucleation and crystallographic path, and both ambient and annealing temperatures influence the nucleation and crystal growth processes. Han et al. [153] revealed that the formation and crystallographic orientations of Ruddlesden–Popper phase perovskites (formula: PEA_2_MA_n-1_Pb_n_Br_3n+1_) are influenced by variations in the precise ambient temperature during solution processing, even in the small range of 21–31 °C; such minute changes in the ambient temperature may occur throughout the day or under different weather conditions. They discovered that more favorable growth occurs in low-*n* phases of the perovskite, particularly for *n* = 1, with an orientation parallel to the substrate. The coexistence of vertical and horizontal orientations and the suppression of the *n* = 1 phase formation possibly result from the heterogeneous nucleation occurring at the top film surface. The crystallization speed test results, absorption spectral analyses, and XRD patterns revealed that this heterogeneous nucleation is stimulated by accelerated solvent evaporation at higher ambient temperatures. The tiny, cube-shaped perovskite crystal grains visible in both AFM and SEM images are an intriguing feature of the perovskite films manufactured at higher ambient temperatures (Figure 10b,c,e,f). The cubic crystals grew larger with increasing temperature, and as a result, the film prepared at 31 °C displayed a corrugated structure, although the huge cubic crystals appeared to partially demolish it. From the device efficiency (Figure 10g–j), it is speculated that 26 °C is the optimal ambient temperature, which shows better light-emitting performance than others.

The solution temperature for a spin-coating step is an important parameter that can influence the nucleation and crystal growth of perovskites. Shargaieva et al. [154] performed GIWAXS measurements to examine the crystallization of methylammonium lead iodide (MAPbI_3_) from a variety of widely used solvents, including DMSO, DMF, NMP, and GBL. Figure 10l shows 2D contour plots of the evolution of azimuthally integrated GIWAXS patterns of MAPbI_3_ thin films processed at 40, 50, 58, 78, and 100 °C as a function of time. These four solvents exhibited different coordination strengths to lead (DMSO > DMF > NMP > GBL) and evaporation rates (DMF > GBL > NMP > DMSO). It was also found that the formation of the MAPbI_3_ perovskite phase competes with that of solvate-intermediate phases at higher solvent-processing temperatures.

Bischak et al. [155] witnessed nucleation and crystal growth during thermally driven transitions to the perovskite phase in a large number of non-perovskite-phase nanowires. They discovered that activated ion diffusion occurs over a thin, yet finite, liquid-like interface and propagates the boundary between the crystal structures, and that both nucleation and growth were characterized by extremely anisotropic kinetics. A solvent engineering strategy [156] was developed to lower the crystallization temperature of MAPbI_3_ single-crystal films (90 °C) and produced higher-quality films with extended carrier lifetimes. Propylene carbonate (PC) with GBL was mixed to reduce the temperature, and it was observed that the nucleation temperature was reduced to 60 °C after mixing PC with GBL from the original 100 °C (without-PC case). Then, with the PC and GBL solvent mixture, the crystal-growth temperature reached 90 °C instead of 130 °C, which was observed in the without-PC case. As a result, large-sized crystals suitable for PSCs were formed. A controlled primary nucleation-aided restricted volume solvent annealing (NR-RVSA) technique [157] was presented with a mixed organic-cation-based perovskite film for developing highly effective (highest PCE: 16.78%) and thermally stable PSCs for flexible substrates. During the critical primary nucleation stage, the NR-RVSA technique increases the degree of supersaturation of the precursor solution, ensuring a uniform perovskite grain dispersion during the fast crystal-development phase. Furthermore, unlike the typical RVSA film, the NR-RVSA perovskite film did not show any residual solvent throughout the secondary crystallization stage, which is driven by thermal annealing, leading to a compact and pinhole-free perovskite film.

## 10. Summary and Outlook

In summary, nucleation and crystallization are the primary deciding factors for processing highly efficient perovskite devices. As discussed, tuning the relationship between nucleation and crystallization aids in realizing controlled crystal growth. Further, the Gibbs free energy is the key parameter that governs the nucleation and subsequent growth of nuclei. More importantly, heterogeneous nucleation occurs frequently, which is discussed in terms of perovskite solution kinetics. Most of the recent studies are focused on crystallization or heterogeneous nucleation. To date, numerous strategies have been reported for regulating nucleation and crystal growth. In this article, we present different strategies, such as changing the perovskite precursor concentration, using the antisolvent dripping technique, and leveraging the solvent or cosolvent effect on the nucleation and growth of crystals, the relationship between the contact angle and homogeneous and heterogeneous nucleation, the effect of surface wettability with changes in the contact angle, the effect of substrate preheating, and the effect of the annealing or ambient temperature. In addition, the effects of grain boundaries and sizes on the interfacial properties of perovskites are also elucidated to obtain a better understanding of the nucleation and crystal growth mechanisms. For single-crystal perovskites, seed-induced crystal growth and substrate-modified heterogeneous nucleation and crystal growth exhibit better optoelectronic performance by changing the interfacial properties. For nanocrystals, perovskite QDs and nanorods show the potential to serve as heterogeneous nucleation sites that facilitate crystal growth with a uniform film morphology. Although substrate preheating and annealing temperatures may also affect the nucleation process, the effect of changes in ambient temperature on crystallization kinetics remains severely under-explored [153].

Many studies so far have shown possible technical strategies to apply nucleation and crystal growth mechanisms for better-quality films and thus high-performance devices. Nevertheless, the understanding of the impact of heterogeneous nucleation on the bulk crystalline properties with respect to the crystallinity and orientation is not yet complete. The precise control and elucidation of hidden factors for controlling the kinetics of nucleation and growth may be required in further work to realize high device performance that is close to the theoretical limit of efficiency with excellent reproducibility. Therefore, there is still room for further research on solution-chemistry-assisted heterogeneous nucleation, as solution chemistry is more conducive to determining bulk crystallinity and provides new avenues for further analyzing perovskite crystal growth and nucleation in the future.

## Data Availability

Not applicable.

## References

[1] Lu H., Krishna A., Zakeeruddin S.M., Grätzel M., Hagfeldt A. (2020). Compositional and Interface Engineering of Organic-Inorganic Lead Halide Perovskite Solar Cells. iScience.

[2] Kojima A., Teshima K., Shirai Y., Miyasaka T. (2009). Organometal Halide Perovskites as Visible-Light Sensitizers for Photovoltaic Cells. J. Am. Chem. Soc..

[3] Lee M.M., Teuscher J., Miyasaka T., Murakami T.N., Snaith H.J. (2012). Efficient Hybrid Solar Cells Based on Meso-Superstructured Organometal Halide Perovskites. Science.

[4] Yoo J.J., Seo G., Chua M.R., Park T.G., Lu Y., Rotermund F., Kim Y.-K., Moon C.S., Jeon N.J., Correa-Baena J.-P. (2021). Efficient perovskite solar cells via improved carrier management. Nature.

[5] Fang T., Wang T., Li X., Dong Y., Bai S., Song J. (2021). Perovskite QLED with an external quantum efficiency of over 21% by modulating electronic transport. Sci. Bull..

[6] Ye J., Byranvand M.M., Martínez C.O., Hoye R.L.Z., Saliba M., Polavarapu L. (2021). Defect Passivation in Lead-Halide Perovskite Nanocrystals and Thin Films: Toward Efficient LEDs and Solar Cells. Angew. Chem. Int. Ed..

[7] Tao P., Liu S.-J., Wong W.-Y. (2020). Phosphorescent Manganese(II) Complexes and Their Emerging Applications. Adv. Opt. Mater..

[8] Fahmi A., Pietsch T., Mendoza C., Cheval N. (2009). Functional hybrid materials. Mater. Today.

[9] Li Y., Yang J., Zhao R., Zhang Y., Wang X., He X., Fu Y., Zhang L. (2022). Design of Organic–Inorganic Hybrid Heterostructured Semiconductors via High-Throughput Materials Screening for Optoelectronic Applications. J. Am. Chem. Soc..

[10] Wu B., Nguyen H.T., Ku Z., Han G., Giovanni D., Mathews N., Fan H.J., Sum T.C. (2016). Discerning the Surface and Bulk Recombination Kinetics of Organic–Inorganic Halide Perovskite Single Crystals. Adv. Energy Mater..

[11] Tan Z.K., Moghaddam R.S., Lai M.L., Docampo P., Higler R., Deschler F., Price M., Sadhanala A., Pazos L.M., Credgington D. (2014). Bright light-emitting diodes based on organometal halide perovskite. Nat. Nanotechnol..

[12] Liu X.K., Xu W., Bai S., Jin Y., Wang J., Friend R.H., Gao F. (2021). Metal halide perovskites for light-emitting diodes. Nat. Mater..

[13] Stoumpos C.C., Kanatzidis M.G. (2015). The Renaissance of Halide Perovskites and Their Evolution as Emerging Semiconductors. Acc. Chem. Res..

[14] Yin W.-J., Yang J.-H., Kang J., Yan Y., Wei S.-H. (2015). Halide perovskite materials for solar cells: A theoretical review. J. Mater. Chem. A.

[15] Jena A.K., Kulkarni A., Miyasaka T. (2019). Halide Perovskite Photovoltaics: Background, Status, and Future Prospects. Chem. Rev..

[16] Hsiao Y.-C., Wu T., Li M., Liu Q., Qin W., Hu B. (2015). Fundamental physics behind high-efficiency organo-metal halide perovskite solar cells. J. Mater. Chem. A.

[17] Wang Y., Zhang Y., Zhang P., Zhang W. (2015). High intrinsic carrier mobility and photon absorption in the perovskite CH_3_NH_3_PbI_3_. Phys. Chem. Chem. Phys..

[18] Kim G.-W., Petrozza A. (2020). Defect Tolerance and Intolerance in Metal-Halide Perovskites. Adv. Energy Mater..

[19] Zhang X., Turiansky M.E., Shen J.-X., Walle C.G.V.d. (2022). Defect tolerance in halide perovskites: A first-principles perspective. J. Appl. Phys..

[20] Adhyaksa G.W.P., Veldhuizen L.W., Kuang Y., Brittman S., Schropp R.E.I., Garnett E.C. (2016). Carrier Diffusion Lengths in Hybrid Perovskites: Processing, Composition, Aging, and Surface Passivation Effects. Chem. Mater..

[21] Zhang D., Cui B.-B., Zhou C., Li L., Chen Y., Zhou N., Xu Z., Li Y., Zhou H., Chen Q. (2017). Reduction of intrinsic defects in hybrid perovskite films via precursor purification. Chem. Commun..

[22] Kim J., Park B.-W., Baek J., Yun J.S., Kwon H.-W., Seidel J., Min H., Coelho S., Lim S., Huang S. (2020). Unveiling the Relationship between the Perovskite Precursor Solution and the Resulting Device Performance. J. Am. Chem. Soc..

[23] Yang W.S., Park B.-W., Jung E.H., Jeon N.J., Kim Y.C., Lee D.U., Shin S.S., Seo J., Kim E.K., Noh J.H. (2017). Iodide management in formamidinium-lead-halide–based perovskite layers for efficient solar cells. Science.

[24] Azmi R., Nurrosyid N., Lee S.-H., Al Mubarok M., Lee W., Hwang S., Yin W., Ahn T.K., Kim T.-W., Ryu D.Y. (2020). Shallow and Deep Trap State Passivation for Low-Temperature Processed Perovskite Solar Cells. ACS Energy Lett..

[25] Bai L., Yao F., Wang R., Liu B., He D., Zhou Q., Wang W., Xu C., Hu X., Chen S. (2022). Ion migration suppression mechanism via 4-sulfobenzoic acid monopotassium salt for 22.7% stable perovskite solar cells. Sci. China Mater..

[26] Yavari M., Ebadi F., Meloni S., Wang Z.S., Yang T.C.-J., Sun S., Schwartz H., Wang Z., Niesen B., Durantini J. (2019). How far does the defect tolerance of lead-halide perovskites range? The example of Bi impurities introducing efficient recombination centers. J. Mater. Chem. A.

[27] Brandt R.E., Stevanović V., Ginley D.S., Buonassisi T. (2015). Identifying defect-tolerant semiconductors with high minority-carrier lifetimes: Beyond hybrid lead halide perovskites. MRS Commun..

[28] Meggiolaro D., Motti S.G., Mosconi E., Barker A.J., Ball J., Andrea Riccardo Perini C., Deschler F., Petrozza A., De Angelis F. (2018). Iodine chemistry determines the defect tolerance of lead-halide perovskites. Energy Environ. Sci..

[29] Kang J., Wang L.-W. (2017). High Defect Tolerance in Lead Halide Perovskite CsPbBr3. J. Phys. Chem. Lett..

[30] Mandal S., Mukherjee S., De C.K., Roy D., Ghosh S., Mandal P.K. (2020). Extent of Shallow/Deep Trap States beyond the Conduction Band Minimum in Defect-Tolerant CsPbBr3 Perovskite Quantum Dot: Control over the Degree of Charge Carrier Recombination. J. Phys. Chem. Lett..

[31] Alharbi E.A., Alyamani A.Y., Kubicki D.J., Uhl A.R., Walder B.J., Alanazi A.Q., Luo J., Burgos-Caminal A., Albadri A., Albrithen H. (2019). Atomic-level passivation mechanism of ammonium salts enabling highly efficient perovskite solar cells. Nat. Commun..

[32] Xu L., Li J., Cai B., Song J., Zhang F., Fang T., Zeng H. (2020). A bilateral interfacial passivation strategy promoting efficiency and stability of perovskite quantum dot light-emitting diodes. Nat. Commun..

[33] Li Y., Wang B., Liu T., Zeng Q., Cao D., Pan H., Xing G. (2022). Interfacial Engineering of PTAA/Perovskites for Improved Crystallinity and Hole Extraction in Inverted Perovskite Solar Cells. ACS Appl. Mater. Interfaces.

[34] Hao M., Bai Y., Zeiske S., Ren L., Liu J., Yuan Y., Zarrabi N., Cheng N., Ghasemi M., Chen P. (2020). Ligand-assisted cation-exchange engineering for high-efficiency colloidal Cs1−xFAxPbI3 quantum dot solar cells with reduced phase segregation. Nat. Energy.

[35] Pan H., Xu X., Liu J., Li X., Zhang H., Huang A., Xiao Z. (2021). Microwave-assisted synthesis of blue-emitting cesium bismuth bromine perovskite nanocrystals without polar solvent. J. Alloys Compd..

[36] Pan A., He B., Fan X., Liu Z., Urban J.J., Alivisatos A.P., He L., Liu Y. (2016). Insight into the Ligand-Mediated Synthesis of Colloidal CsPbBr3 Perovskite Nanocrystals: The Role of Organic Acid, Base, and Cesium Precursors. ACS Nano.

[37] Yang Y., Yang M., Moore D.T., Yan Y., Miller E.M., Zhu K., Beard M.C. (2017). Top and bottom surfaces limit carrier lifetime in lead iodide perovskite films. Nat. Energy.

[38] McMeekin D.P., Wang Z., Rehman W., Pulvirenti F., Patel J.B., Noel N.K., Johnston M.B., Marder S.R., Herz L.M., Snaith H.J. (2017). Crystallization Kinetics and Morphology Control of Formamidinium–Cesium Mixed-Cation Lead Mixed-Halide Perovskite via Tunability of the Colloidal Precursor Solution. Adv. Mater..

[39] Zhang S., Wu S., Chen R., Chen W., Huang Y., Zhu H., Yang Z., Chen W. (2019). Controlling Orientation Diversity of Mixed Ion Perovskites: Reduced Crystal Microstrain and Improved Structural Stability. J. Phys. Chem. Lett..

[40] Zhao Y., Zhou W., Han Z., Yu D., Zhao Q. (2021). Effects of ion migration and improvement strategies for the operational stability of perovskite solar cells. Phys. Chem. Chem. Phys..

[41] Shao Y., Fang Y., Li T., Wang Q., Dong Q., Deng Y., Yuan Y., Wei H., Wang M., Gruverman A. (2016). Grain boundary dominated ion migration in polycrystalline organic–inorganic halide perovskite films. Energy Environ. Sci..

[42] Eames C., Frost J.M., Barnes P.R.F., O’Regan B.C., Walsh A., Islam M.S. (2015). Ionic transport in hybrid lead iodide perovskite solar cells. Nat. Commun..

[43] Sun C., Jiang Y., Cui M., Qiao L., Wei J., Huang Y., Zhang L., He T., Li S., Hsu H.-Y. (2021). High-performance large-area quasi-2D perovskite light-emitting diodes. Nat. Commun..

[44] Yang Z., Dou J., Kou S., Dang J., Ji Y., Yang G., Wu W.-Q., Kuang D.-B., Wang M. (2020). Multifunctional Phosphorus-Containing Lewis Acid and Base Passivation Enabling Efficient and Moisture-Stable Perovskite Solar Cells. Adv. Funct. Mater..

[45] Garai R., Gupta R.K., Tanwar A.S., Hossain M., Iyer P.K. (2021). Conjugated Polyelectrolyte-Passivated Stable Perovskite Solar Cells for Efficiency Beyond 20%. Chem. Mater..

[46] Chao L., Niu T., Xia Y., Chen Y., Huang W. (2021). Ionic Liquid for Perovskite Solar Cells: An Emerging Solvent Engineering Technology. Acc. Mater. Res..

[47] Yun J.S., Ho-Baillie A., Huang S., Woo S.H., Heo Y., Seidel J., Huang F., Cheng Y.-B., Green M.A. (2015). Benefit of Grain Boundaries in Organic–Inorganic Halide Planar Perovskite Solar Cells. J. Phys. Chem. Lett..

[48] Park M.-H., Park J., Lee J., So H.S., Kim H., Jeong S.-H., Han T.-H., Wolf C., Lee H., Yoo S. (2019). Efficient Perovskite Light-Emitting Diodes Using Polycrystalline Core–Shell-Mimicked Nanograins. Adv. Funct. Mater..

[49] Liu W., Liu N., Ji S., Hua H., Ma Y., Hu R., Zhang J., Chu L., Li X.a., Huang W. (2020). Perfection of Perovskite Grain Boundary Passivation by Rhodium Incorporation for Efficient and Stable Solar Cells. Nano-Micro Lett..

[50] Hu X., Meng X., Yang X., Huang Z., Xing Z., Li P., Tan L., Su M., Li F., Chen Y. (2021). Cementitious grain-boundary passivation for flexible perovskite solar cells with superior environmental stability and mechanical robustness. Sci. Bull..

[51] Sánchez S., Pfeifer L., Vlachopoulos N., Hagfeldt A. (2021). Rapid hybrid perovskite film crystallization from solution. Chem. Soc. Rev..

[52] Kumar J., Srivastava P., Bag M. (2022). Advanced Strategies to Tailor the Nucleation and Crystal Growth in Hybrid Halide Perovskite Thin Films. Front. Chem..

[53] Kim H.-S., Park N.-G. (2020). Importance of tailoring lattice strain in halide perovskite crystals. NPG Asia Mater..

[54] Thanh N.T.K., Maclean N., Mahiddine S. (2014). Mechanisms of Nucleation and Growth of Nanoparticles in Solution. Chem. Rev..

[55] McGinty J., Yazdanpanah N., Price C., ter Horst J.H., Sefcik J. (2020). CHAPTER 1 Nucleation and Crystal Growth in Continuous Crystallization. The Handbook of Continuous Crystallization.

[56] Liu D., Zhou W., Tang H., Fu P., Ning Z. (2018). Supersaturation controlled growth of MAFAPbI3 perovskite film for high efficiency solar cells. Sci. China Chem..

[57] Hu H., Singh M., Wan X., Tang J., Chu C.-W., Li G. (2020). Nucleation and crystal growth control for scalable solution-processed organic–inorganic hybrid perovskite solar cells. J. Mater. Chem. A.

[58] Taqieddin A., Allshouse M.R., Alshawabkeh A.N. (2018). Editors’ Choice—Critical Review—Mathematical Formulations of Electrochemically Gas-Evolving Systems. J. Electrochem. Soc..

[59] Gao Q., Qi J., Chen K., Xia M., Hu Y., Mei A., Han H. (2022). Halide Perovskite Crystallization Processes and Methods in Nanocrystals, Single Crystals, and Thin Films. Adv. Mater..

[60] Gebremichael Z.T., Ugokwe C., Alam S., Stumpf S., Diegel M., Schubert U.S., Hoppe H. (2022). How varying surface wettability of different PEDOT:PSS formulations and their mixtures affects perovskite crystallization and the efficiency of inverted perovskite solar cells. RSC Adv..

[61] Xu H. (2020). A brief review on the moisture stability for perovskite solar cells. IOP Conf. Ser. Earth Environ. Sci..

[62] Meng X., Wang Y., Lin J., Liu X., He X., Barbaud J., Wu T., Noda T., Yang X., Han L. (2020). Surface-Controlled Oriented Growth of FASnI3 Crystals for Efficient Lead-free Perovskite Solar Cells. Joule.

[63] Li B., Deng J., Smith J.A., Caprioglio P., Ji K., Luo D., McGettrick J.D., Jayawardena K.D.G.I., Kilbride R.C., Ren A. (2022). Suppressing Interfacial Recombination with a Strong-Interaction Surface Modulator for Efficient Inverted Perovskite Solar Cells. Adv. Energy Mater..

[64] Khaleel O.A., Ahmed D.S. (2022). Introduction of γ-butyrolactone (GBL) solvent to assist perovskite crystallization and develop stable and efficient perovskite solar cells. Opt. Mater..

[65] Pylnev M., Barbisan A.M., Wei T.-C. (2021). Effect of wettability of substrate on metal halide perovskite growth. Appl. Surf. Sci..

[66] Yao H., Peng G., Li Z., Wang Q., Xu Y., Ma B., Lei Y., Wang G., Wang Q., Ci Z. (2022). Fine coverage and uniform phase distribution in 2D (PEA)2Cs3Pb4I13 solar cells with a record efficiency beyond 15%. Nano Energy.

[67] Jung E.D., Harit A.K., Kim D.H., Jang C.H., Park J.H., Cho S., Song M.H., Woo H.Y. (2020). Multiply Charged Conjugated Polyelectrolytes as a Multifunctional Interlayer for Efficient and Scalable Perovskite Solar Cells. Adv. Mater..

[68] Bi C., Wang Q., Shao Y., Yuan Y., Xiao Z., Huang J. (2015). Non-wetting surface-driven high-aspect-ratio crystalline grain growth for efficient hybrid perovskite solar cells. Nat. Commun..

[69] Wang G., Liu J., Lei M., Zhang W., Zhu G. (2020). Optimizing the substrate pre-heating and post-annealing temperatures for fabricating high-performance carbon-based CsPbIBr2 inorganic perovskite solar cells. Electrochim. Acta.

[70] Huang K., Li H., Zhang C., Gao Y., Liu T., Zhang J., Gao Y., Peng Y., Ding L., Yang J. (2019). Highly Efficient Perovskite Solar Cells Processed Under Ambient Conditions Using In Situ Substrate-Heating-Assisted Deposition. Sol. RRL.

[71] Wei H., Tang Y., Feng B., You H. (2017). Importance of PbI2 morphology in two-step deposition of CH_3_NH_3_PbI_3_ for high-performance perovskite solar cells*. Chin. Phys. B.

[72] Srivastava P., Parhi A.P., Ranjan R., Satapathi S., Bag M. (2018). Temperature Assisted Nucleation and Growth To Optimize Perovskite Morphology at Liquid Interface: A Study by Electrochemical Impedance Spectroscopy. ACS Appl. Energy Mater..

[73] Min H., Hu J., Xu Z., Liu T., Khan S.-U.-Z., Roh K., Loo Y.-L., Rand B.P. (2022). Hot-Casting-Assisted Liquid Additive Engineering for Efficient and Stable Perovskite Solar Cells. Adv. Mater..

[74] Chen J., Zuo L., Zhang Y., Lian X., Fu W., Yan J., Li J., Wu G., Li C.-Z., Chen H. (2018). High-Performance Thickness Insensitive Perovskite Solar Cells with Enhanced Moisture Stability. Adv. Energy Mater..

[75] Hu S., Yang X., Yang B., Zhang Y., Li H., Sheng C. (2021). Excitonic Solar Cells Using 2D Perovskite of (BA)2(FA)2Pb3I10. J. Phys. Chem. C.

[76] Chang C.-Y., Huang Y.-C., Tsao C.-S., Su W.-F. (2016). Formation Mechanism and Control of Perovskite Films from Solution to Crystalline Phase Studied by in Situ Synchrotron Scattering. ACS Appl. Mater. Interfaces.

[77] Zhang X., Munir R., Xu Z., Liu Y., Tsai H., Nie W., Li J., Niu T., Smilgies D.-M., Kanatzidis M.G. (2018). Phase Transition Control for High Performance Ruddlesden–Popper Perovskite Solar Cells. Adv. Mater..

[78] Fan Y., Fang J., Chang X., Tang M.-C., Barrit D., Xu Z., Jiang Z., Wen J., Zhao H., Niu T. (2019). Scalable Ambient Fabrication of High-Performance CsPbI2Br Solar Cells. Joule.

[79] Tang S., Deng Y., Zheng X., Bai Y., Fang Y., Dong Q., Wei H., Huang J. (2017). Composition Engineering in Doctor-Blading of Perovskite Solar Cells. Adv. Energy Mater..

[80] Li T., Rui Y., Wang X., Shi J., Wang Y., Yang J., Zhang Q. (2021). Grain Size and Interface Modification via Cesium Carbonate Post-Treatment for Efficient SnO2-Based Planar Perovskite Solar Cells. ACS Appl. Energy Mater..

[81] Kim H., Hong J., Kim C., Shin E.-Y., Lee M., Noh Y.-Y., Park B., Hwang I. (2018). Impact of Hydroxyl Groups Boosting Heterogeneous Nucleation on Perovskite Grains and Photovoltaic Performances. J. Phys. Chem. C.

[82] Degani M., An Q., Albaladejo-Siguan M., Hofstetter Y.J., Cho C., Paulus F., Grancini G., Vaynzof Y. (2021). 23.7% Efficient inverted perovskite solar cells by dual interfacial modification. Sci. Adv..

[83] Liao K., Xie L., Cui Y., Wang S., Li C., Wang A., Deng X., Xiang Y., Ding L., Hao F. (2020). Aqueous solvent-regulated crystallization and interfacial modification in perovskite solar cells with enhanced stability and performance. J. Power Sources.

[84] Liu Q.-W., Yuan S., Sun S.-Q., Luo W., Zhang Y.-J., Liao L.-S., Fung M.-K. (2019). Interfacial engineering for highly efficient quasi-two dimensional organic–inorganic hybrid perovskite light-emitting diodes. J. Mater. Chem. C.

[85] Tan Y., Chang X., Zhong J.-X., Feng W., Yang M., Tian T., Gong L., Wu W.-Q. (2022). Chemical Linkage and Passivation at Buried Interface for Thermally Stable Inverted Perovskite Solar Cells with Efficiency over 22%. CCS Chem..

[86] Kim S., Jeong J.-E., Hong J., Lee K., Lee M.J., Woo H.Y., Hwang I. (2020). Improved Interfacial Crystallization by Synergic Effects of Precursor Solution Stoichiometry and Conjugated Polyelectrolyte Interlayer for High Open-Circuit Voltage of Perovskite Photovoltaic Diodes. ACS Appl. Mater. Interfaces.

[87] Liu Q., Lv P., Wang Y., Zhu Y., Hu M., Huang F., Cheng Y.-B., Lu J. (2022). Impact of Nickel Oxide/Perovskite Interfacial Contact on the Crystallization and Photovoltaic Performance of Perovskite Solar Cells. Sol. RRL.

[88] Xu H., Miao Y., Wei N., Chen H., Qin Z., Liu X., Wang X., Qi Y., Zhang T., Zhao Y. (2022). CsI Enhanced Buried Interface for Efficient and UV-Robust Perovskite Solar Cells. Adv. Energy Mater..

[89] Xiong Z., Chen X., Zhang B., Odunmbaku G.O., Ou Z., Guo B., Yang K., Kan Z., Lu S., Chen S. (2022). Simultaneous Interfacial Modification and Crystallization Control by Biguanide Hydrochloride for Stable Perovskite Solar Cells with PCE of 24.4%. Adv. Mater..

[90] Tong G., Ono L.K., Liu Y., Zhang H., Bu T., Qi Y. (2021). Up-Scalable Fabrication of SnO2 with Multifunctional Interface for High Performance Perovskite Solar Modules. Nano-Micro Lett..

[91] Ma L., Yan Z., Zhou X., Pi Y., Du Y., Huang J., Wang K., Wu K., Zhuang C., Han X. (2021). A polymer controlled nucleation route towards the generalized growth of organic-inorganic perovskite single crystals. Nat. Commun..

[92] Ren Y., Hao Y., Zhang N., Arain Z., Mateen M., Sun Y., Shi P., Cai M., Dai S. (2020). Exploration of polymer-assisted crystallization kinetics in CsPbBr3 all-inorganic solar cell. Chem. Eng. J..

[93] Nasti G., Aldamasy M.H., Flatken M.A., Musto P., Matczak P., Dallmann A., Hoell A., Musiienko A., Hempel H., Aktas E. (2022). Pyridine Controlled Tin Perovskite Crystallization. ACS Energy Lett..

[94] Zhao H., Li T., Li J., Li Q., Wang S., Zheng C., Li J., Li M., Zhang Y., Yao J. (2022). Excess polymer-assisted crystal growth method for high-performance perovskite photodetectors. J. Alloys Compd..

[95] Cao Q., Li Y., Zhang H., Yang J., Han J., Xu T., Wang S., Wang Z., Gao B., Zhao J. (2021). Efficient and stable inverted perovskite solar cells with very high fill factors via incorporation of star-shaped polymer. Sci. Adv..

[96] Mei Y., Sun M., Liu H., Li X., Wang S. (2021). Polymer additive assisted crystallization of perovskite films for high-performance solar cells. Org. Electron..

[97] Feng W., Zhao Y., Lin K., Lu J., Liang Y., Liu K., Xie L., Tian C., Lyu T., Wei Z. (2022). Polymer-Assisted Crystal Growth Regulation and Defect Passivation for Efficient Perovskite Light-Emitting Diodes. Adv. Funct. Mater..

[98] Guo J., Wang K., Liu T., Wei Q., Mei S., Yu X., Tang Z., Xing G., Hong G. (2021). Suppressing the defects in cesium-based perovskites via polymeric interlayer assisted crystallization control. J. Mater. Chem. A.

[99] Du S., Yang J., Qu S., Lan Z., Sun T., Dong Y., Shang Z., Liu D., Yang Y., Yan L. (2022). Impact of Precursor Concentration on Perovskite Crystallization for Efficient Wide-Bandgap Solar Cells. Materials.

[100] Ezike S.C., Ahmed A.D., Obodo R.M., Salawu M.A. (2022). Perovskite precursor concentration for enhanced recombination suppression in perovskite solar cells. Hybrid Adv..

[101] Jiang S., Sheng Y., Hu Y., Rong Y., Mei A., Han H. (2020). Influence of precursor concentration on printable mesoscopic perovskite solar cells. Front. Opt..

[102] Wieghold S., Correa-Baena J.-P., Nienhaus L., Sun S., Shulenberger K.E., Liu Z., Tresback J.S., Shin S.S., Bawendi M.G., Buonassisi T. (2018). Precursor Concentration Affects Grain Size, Crystal Orientation, and Local Performance in Mixed-Ion Lead Perovskite Solar Cells. ACS Appl. Energy Mater..

[103] Yao Z., Wang W., Shen H., Zhang Y., Luo Q., Yin X., Dai X., Li J., Lin H. (2017). CH_3_NH_3_PbI_3_ grain growth and interfacial properties in meso-structured perovskite solar cells fabricated by two-step deposition. Sci. Technol. Adv. Mater..

[104] Sanni D.M., Yerramilli A.S., Ntsoenzok E., Adeniji S.A., Oyelade O.V., Koech R.K., Fashina A.A., Alford T.L. (2021). Impact of precursor concentration on the properties of perovskite solar cells obtained from the dehydrated lead acetate precursors. J. Vac. Sci. Technol. A.

[105] Zhang Z.L., Men B.Q., Liu Y.F., Gao H.P., Mao Y.L. (2017). Effects of precursor solution composition on the performance and I-V hysteresis of perovskite solar cells based on CH_3_NH_3_PbI_3-x_Cl_x_. Nanoscale Res. Lett..

[106] Jung M., Ji S.-G., Kim G., Seok S.I. (2019). Perovskite precursor solution chemistry: From fundamentals to photovoltaic applications. Chem. Soc. Rev..

[107] Ghosh S., Mishra S., Singh T. (2020). Antisolvents in Perovskite Solar Cells: Importance, Issues, and Alternatives. Adv. Mater. Interfaces.

[108] Babayigit A., D’Haen J., Boyen H.-G., Conings B. (2018). Gas Quenching for Perovskite Thin Film Deposition. Joule.

[109] Brinkmann K.O., He J., Schubert F., Malerczyk J., Kreusel C., van gen Hassend F., Weber S., Song J., Qu J., Riedl T. (2019). Extremely Robust Gas-Quenching Deposition of Halide Perovskites on Top of Hydrophobic Hole Transport Materials for Inverted (p–i–n) Solar Cells by Targeting the Precursor Wetting Issue. ACS Appl. Mater. Interfaces.

[110] Hou T., Zhang M., Yu W., Wang X., Gu Z., Chen Q., Lan L., Sun X., Huang Y., Zheng B. (2022). Low-pressure accessible gas-quenching for absolute methylammonium-free perovskite solar cells. J. Mater. Chem. A.

[111] Szostak R., Sanchez S., Marchezi P.E., Marques A.S., Silva J.C., Holanda M.S., Hagfeldt A., Tolentino H.C.N., Nogueira A.F. (2021). Revealing the Perovskite Film Formation Using the Gas Quenching Method by In Situ GIWAXS: Morphology, Properties, and Device Performance. Adv. Funct. Mater..

[112] Yang J., Yu H., Wu S., Cai C., Gao J., Lu X., Gao X., Shui L., Wu S., Liu J.-M. (2022). A Mixed Antisolvent-Assisted Crystallization Strategy for Efficient All-Inorganic CsPbIBr2 Perovskite Solar Cells by a Low-Temperature Process. ACS Appl. Energy Mater..

[113] Samadpour M., Golchini A., Abdizadeh K., Heydari M., Forouzandeh M., Saki Z., Taghavinia N. (2021). Modified Antisolvent Method for Improving the Performance and Stability of Triple-Cation Perovskite Solar Cells. ACS Omega.

[114] Dahal B., Li W. (2022). Configuration of Methylammonium Lead Iodide Perovskite Solar Cell and its Effect on the Device’s Performance: A Review. Adv. Mater. Interfaces.

[115] Xiao M., Zhao L., Geng M., Li Y., Dong B., Xu Z., Wan L., Li W., Wang S. (2018). Selection of an anti-solvent for efficient and stable cesium-containing triple cation planar perovskite solar cells. Nanoscale.

[116] Lewis A.E., Zhang Y., Gao P., Nazeeruddin M.K. (2017). Unveiling the Concentration-Dependent Grain Growth of Perovskite Films from One- and Two-Step Deposition Methods: Implications for Photovoltaic Application. ACS Appl. Mater. Interfaces.

[117] Liu C., Yang Y., Syzgantseva O.A., Ding Y., Syzgantseva M.A., Zhang X., Asiri A.M., Dai S., Nazeeruddin M.K. (2020). α-CsPbI3 Bilayers via One-Step Deposition for Efficient and Stable All-Inorganic Perovskite Solar Cells. Adv. Mater..

[118] Fang F., Chen J., Wu G., Chen H. (2018). Highly efficient perovskite solar cells fabricated by simplified one-step deposition method with non-halogenated anti-solvents. Org. Electron..

[119] Taylor A.D., Sun Q., Goetz K.P., An Q., Schramm T., Hofstetter Y., Litterst M., Paulus F., Vaynzof Y. (2021). A general approach to high-efficiency perovskite solar cells by any antisolvent. Nat. Commun..

[120] Cao X., Zhi L., Jia Y., Li Y., Zhao K., Cui X., Ci L., Zhuang D., Wei J. (2019). A Review of the Role of Solvents in Formation of High-Quality Solution-Processed Perovskite Films. ACS Appl. Mater. Interfaces.

[121] Wang W.-T., Das S.K., Tai Y. (2017). Fully Ambient-Processed Perovskite Film for Perovskite Solar Cells: Effect of Solvent Polarity on Lead Iodide. ACS Appl. Mater. Interfaces.

[122] Jiang J., Vicent-Luna J.M., Tao S. (2022). The role of solvents in the formation of methylammonium lead triiodide perovskite. J. Energy Chem..

[123] Chen J., Xiong Y., Rong Y., Mei A., Sheng Y., Jiang P., Hu Y., Li X., Han H. (2016). Solvent effect on the hole-conductor-free fully printable perovskite solar cells. Nano Energy.

[124] Ahn N., Son D.-Y., Jang I.-H., Kang S.M., Choi M., Park N.-G. (2015). Highly Reproducible Perovskite Solar Cells with Average Efficiency of 18.3% and Best Efficiency of 19.7% Fabricated via Lewis Base Adduct of Lead(II) Iodide. J. Am. Chem. Soc..

[125] Arain Z., Liu C., Yang Y., Mateen M., Ren Y., Ding Y., Liu X., Ali Z., Kumar M., Dai S. (2019). Elucidating the dynamics of solvent engineering for perovskite solar cells. Sci. China Mater..

[126] Chen A.Z., Shiu M., Deng X., Mahmoud M., Zhang D., Foley B.J., Lee S.-H., Giri G., Choi J.J. (2019). Understanding the Formation of Vertical Orientation in Two-dimensional Metal Halide Perovskite Thin Films. Chem. Mater..

[127] Li H., Xu Y., Ramakrishnan S., Zhang Y., Cotlet M., Xu T.L., Yu Q. (2022). Pseudo-halide anion engineering for efficient quasi-2D Ruddlesden-Popper tin perovskite solar cells. Cell Rep. Phys. Sci..

[128] Kong L., Luo Y., Turyanska L., Zhang T., Zhang Z., Xing G., Yang Y., Zhang C., Yang X. (2023). A Spacer Cation Assisted Nucleation and Growth Strategy Enables Efficient and High-Luminance Quasi-2D Perovskite LEDs. Adv. Funct. Mater..

[129] Lee J., Jang G., Ma S., Lee C.U., Son J., Jeong W., Moon J. (2022). Universal Bifacial Stamping Approach Enabling Reverse-Graded Ruddlesden-Popper 2D Perovskite Solar Cells. Small.

[130] Liu T., Wei Q., Cai S., He B., Su Z., Zhang Z., Zhang Y., Zhou H., Wang G., Huang Y. (2022). Self-Assembled Bilayer Microstructure Improves Quasi-2D Perovskite Light-Emitting Diodes. Chem. Mater..

[131] Guo Z., Zhang Y., Wang B., Wang L., Zhou N., Qiu Z., Li N., Chen Y., Zhu C., Xie H. (2021). Promoting Energy Transfer via Manipulation of Crystallization Kinetics of Quasi-2D Perovskites for Efficient Green Light-Emitting Diodes. Adv. Mater..

[132] Zheng G., Zhu C., Ma J., Zhang X., Tang G., Li R., Chen Y., Li L., Hu J., Hong J. (2018). Manipulation of facet orientation in hybrid perovskite polycrystalline films by cation cascade. Nat. Commun..

[133] Yang Y., Wu L., Hao X., Tang Z., Lai H., Zhang J., Wang W., Feng L. (2019). Beneficial effects of potassium iodide incorporation on grain boundaries and interfaces of perovskite solar cells. RSC Adv..

[134] Zhang J., Yang J., Dai R., Sheng W., Su Y., Zhong Y., Li X., Tan L., Chen Y. (2022). Elimination of Interfacial Lattice Mismatch and Detrimental Reaction by Self-Assembled Layer Dual-Passivation for Efficient and Stable Inverted Perovskite Solar Cells. Adv. Energy Mater..

[135] Kepenekian M., Traore B., Blancon J.-C., Pedesseau L., Tsai H., Nie W., Stoumpos C.C., Kanatzidis M.G., Even J., Mohite A.D. (2018). Concept of Lattice Mismatch and Emergence of Surface States in Two-dimensional Hybrid Perovskite Quantum Wells. Nano Lett..

[136] Qin M., Li Y., Yang Y., Chan P.F., Li S., Qin Z., Guo X., Shu L., Zhu Y., Fan Z. (2022). Regulating the Crystallization Kinetics and Lattice Strain of Lead-Free Perovskites with Perovskite Quantum Dots. ACS Energy Lett..

[137] Cho Y., Jung H.R., Jo W. (2022). Halide perovskite single crystals: Growth, characterization, and stability for optoelectronic applications. Nanoscale.

[138] Abbas M., Zeng L., Guo F., Rauf M., Yuan X.-C., Cai B. (2020). A Critical Review on Crystal Growth Techniques for Scalable Deposition of Photovoltaic Perovskite Thin Films. Materials.

[139] Di H., Jiang W., Sun H., Zhao C., Liao F., Zhao Y. (2020). Effects of ITO Substrate Hydrophobicity on Crystallization and Properties of MAPbBr3 Single-Crystal Thin Films. ACS Omega.

[140] Pratheek M., Chandra G.K., Predeep P. (2022). Ultrathin single-crystalline perovskites: Toward large area wafers. J. Cryst. Growth.

[141] Deng Y.-H., Yang Z.-Q., Ma R.-M. (2020). Growth of centimeter-scale perovskite single-crystalline thin film via surface engineering. Nano Converg..

[142] Chen Z., Dong Q., Liu Y., Bao C., Fang Y., Lin Y., Tang S., Wang Q., Xiao X., Bai Y. (2017). Thin single crystal perovskite solar cells to harvest below-bandgap light absorption. Nat. Commun..

[143] Xiao M., Gu S., Zhu P., Tang M., Zhu W., Lin R., Chen C., Xu W., Yu T., Zhu J. (2018). Tin-Based Perovskite with Improved Coverage and Crystallinity through Tin-Fluoride-Assisted Heterogeneous Nucleation. Adv. Opt. Mater..

[144] Liu Y., Yang Z., Cui D., Ren X., Sun J., Liu X., Zhang J., Wei Q., Fan H., Yu F. (2015). Two-Inch-Sized Perovskite CH_3_NH_3_PbX_3_ (X = Cl, Br, I) Crystals: Growth and Characterization. Adv. Mater..

[145] Gu Z., Huang Z., Li C., Li M., Song Y. (2018). A general printing approach for scalable growth of perovskite single-crystal films. Sci. Adv..

[146] Wang P., Xie J., Xiao K., Hu H., Cui C., Qiang Y., Lin P., Arivazhagan V., Xu L., Yang Z. (2018). CH_3_NH_3_PbBr_3_ Quantum Dot-Induced Nucleation for High Performance Perovskite Light-Emitting Solar Cells. ACS Appl. Mater. Interfaces.

[147] Li X., Zhang K., Li J., Chen J., Wu Y., Liu K., Song J., Zeng H. (2018). Heterogeneous Nucleation toward Polar-Solvent-Free, Fast, and One-Pot Synthesis of Highly Uniform Perovskite Quantum Dots for Wider Color Gamut Display. Adv. Mater. Interfaces.

[148] Yao Y., Hang P., Wang P., Xu L., Cui C., Xie J., Xiao K., Li G., Lin P., Liu S. (2020). CsPbBr3 quantum dots assisted crystallization of solution-processed perovskite films with preferential orientation for high performance perovskite solar cells. Nanotechnology.

[149] Zhang Z., Lamers N., Sun C., Hetherington C., Scheblykin I.G., Wallentin J. (2022). Free-Standing Metal Halide Perovskite Nanowire Arrays with Blue-Green Heterostructures. Nano Lett..

[150] Zhang Y., Yang H., Chen M., Padture N.P., Chen O., Zhou Y. (2019). Fusing Nanowires into Thin Films: Fabrication of Graded-Heterojunction Perovskite Solar Cells with Enhanced Performance. Adv. Energy Mater..

[151] Spina M., Bonvin E., Sienkiewicz A., Náfrádi B., Forró L., Horváth E. (2016). Controlled growth of CH_3_NH_3_PbI_3_ nanowires in arrays ofopen nanofluidic channels. Sci. Rep..

[152] Xu Z., Gong Y., Wang J., Ma Z., Yu R., Yang J., Liu Y., Guo Q., Zhou E., Tan Z.a. (2023). Carbon nanofibers fabricated via electrospinning to guide crystalline orientation for stable perovskite solar cells with efficiency over 24%. Chem. Eng. J..

[153] Han Y., Wang J., Bischak C.G., Kim S., Lee K., Shin D., Lee M.J., Ginger D.S., Hwang I. (2020). Significance of Ambient Temperature Control for Highly Reproducible Layered Perovskite Light-Emitting Diodes. ACS Photonics.

[154] Shargaieva O., Näsström H., Li J., Többens D.M., Unger E.L. (2021). Temperature-Dependent Crystallization Mechanisms of Methylammonium Lead Iodide Perovskite From Different Solvents. Front. Energy Res..

[155] Bischak C.G., Lai M., Fan Z., Lu D., David P., Dong D., Chen H., Etman A.S., Lei T., Sun J. (2020). Liquid-like Interfaces Mediate Structural Phase Transitions in Lead Halide Perovskites. Matter.

[156] Alsalloum A.Y., Turedi B., Zheng X., Mitra S., Zhumekenov A.A., Lee K.J., Maity P., Gereige I., AlSaggaf A., Roqan I.S. (2020). Low-Temperature Crystallization Enables 21.9% Efficient Single-Crystal MAPbI3 Inverted Perovskite Solar Cells. ACS Energy Lett..

[157] Mahmud M.A., Elumalai N.K., Upama M.B., Wang D., Haque F., Wright M., Xu C., Uddin A. (2017). Controlled nucleation assisted restricted volume solvent annealing for stable perovskite solar cells. Sol. Energy Mater. Sol. Cells.

